# Evolution and development of the adelphophagic, intracapsular Schmidt’s larva of the nemertean *Lineus ruber*

**DOI:** 10.1186/s13227-015-0023-5

**Published:** 2015-09-28

**Authors:** José M. Martín-Durán, Bruno C. Vellutini, Andreas Hejnol

**Affiliations:** Sars International Centre for Marine Molecular Biology, University of Bergen, Thormøhlensgate 55, 5008 Bergen, Norway

**Keywords:** Nemertea, *Lineus ruber*, Development, Larva, Adelphophagy, Intracapsular, Metamorphosis, Gastrulation, Germ layers, Imaginal disc

## Abstract

**Background:**

The life cycle of many animals includes a larval stage, which has diversified into an astonishing variety of ecological strategies. The Nemertea is a group of spiralians that exhibits a broad diversity of larval forms, including the iconic pilidium. A pelagic planktotrophic pilidium is the ancestral form in the Pilidiophora, but several lineages exhibit deviations of this condition, mostly as a transition to pelagic lecithotrophy. The most extreme case occurs, however, in the Pilidiophoran *Lineus ruber*, which exhibits an adelphophagic intracapsular pilidium, the so-called Schmidt’s larva.

**Results:**

We combined confocal laser scanning microscopy and gene expression studies to characterize the development and metamorphosis of the Schmidt’s larva of *L. ruber*. The larva forms after gastrulation, and comprises a thin epidermis, a proboscis rudiment and two pairs of imaginal discs from which the juvenile will develop. The cells internalized during gastrulation form a blind gut and the blastopore gives rise to the mouth of the larva and juvenile. The Schmidt’s larva eats other siblings that occupy the same egg capsule, accumulating nutrients for the juvenile. A gradual metamorphosis involves the differentiation of the juvenile cell types from the imaginal discs and the shedding of the larval epidermis. The expression of evolutionarily conserved anterior (*foxQ2*, *six3/6*, *gsc*, *otx*), endomesodermal (*foxA*, *GATA456*-*a*, *twi*-*a*) and posterior (*evx*, *cdx*) markers demonstrate that the juvenile retains the molecular patterning of the Schmidt’s larva. After metamorphosis, the juveniles stay over 20 days within the egg masses, until they are fully mature and hatch.

**Conclusions:**

The evolution of the intracapsular Schmidt’s larva involved the loss of the typical feeding structures of the planktotrophic pilidium and a precocious formation of the imaginal discs, as also observed in other pelagic lecithotrophic forms. However, no special adaptations are observed related to adelphophagy. As in planktotrophic pilidium, the molecular mechanism patterning the juvenile is only active in the imaginal discs and not during the early development of the larva, suggesting two separate molecular programs during nemertean embryogenesis. Our results illuminate the diversification of larval forms in the Pilidiophora and Nemertea, and thus on the developmental mechanisms underlying metazoan larval evolution.

**Electronic supplementary material:**

The online version of this article (doi:10.1186/s13227-015-0023-5) contains supplementary material, which is available to authorized users.

## Background

**Fig. 1:**
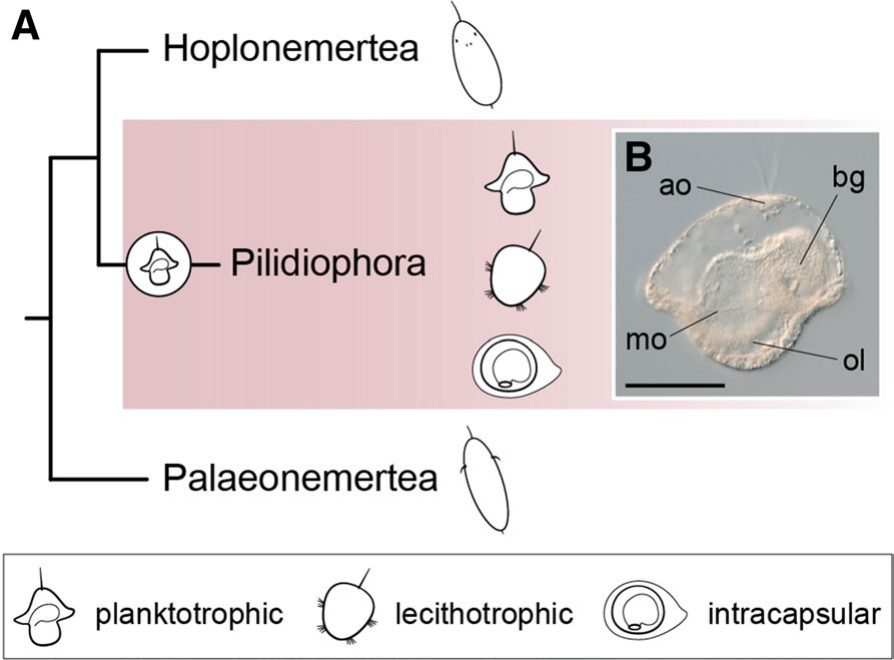
The Nemertea and the diversity of larval forms. **A** The Nemertea comprises three major monophyletic groups, namely the Palaeonemertea, the Hoplonemertea and the Pilidiophora [99, 100]. The Palaeonemertea and Hoplonemertea show the so-called planuliform larvae, while the Pilidiophora shows as ancestral trait the presence of a planktotrophic pilidium larva (shown in **B**). This larva is present in the majority of lineages, although several groups exhibit several specializations, such as a shift towards lecithotrophic and/or encapsulated development. **B** Planktotrophic pilidium larva of *Lineus longissimus*. The typical pilidium has a *hat-like* shape, with an aboral apical organ and a ventral mouth surrounded by two long oral lobes. Internally, the larva has a blind gut, and as it matures, the imaginal discs that will form the adult grow inside. **B** Is a lateral view. Drawings are not to scale. The drawings of the planuliform larva of the Palaeonemertea and Hoplonemertea are adapted from [40]. *ao* apical organ, *bg* blind gut, *mo* mouth, *ol* oral lobes. *Scale bar* in **B**, 100 μm

Nemerteans (“ribbon worms”) are a group of mostly marine predatory worms that exhibit a broad diversity of larval forms and life cycles [1–3]. Phylogenetically, this group is well nested within the Spiralia [4–7], and closely related to the Lophophorate taxa (brachiopods, phoronids and bryozoans) [5, 8–11]. Similar to many other members of the Spiralia, nemerteans exhibit quartet spiral cleavage [12–16], a highly stereotypical mode of development. After cleavage and gastrulation, most nemertean embryos develop into a larval stage, which can be either of the planuliform type—in the Hoplonemertea and Palaeonemertea—or of the pilidium type—in the Pilidiophora [2, 17] (Fig. 1A). In the planuliform larvae, the adult/juvenile body is largely defined [18–20]. They can be planktotrophic or lecithotrophic [2], and the transition into the benthic adult is gradual and without a dramatic metamorphosis [18, 21]. However, planuliform larvae sometimes possess tissues and organs that are discarded during maturation [18, 19, 21]. Pilidium larvae exhibit, on the contrary, an archetypical maximally indirect development. The body plan of the pilidium larva is completely different from the adult morphology, and the transition between these two stages involves a catastrophic metamorphosis [22]. Pilidium larvae often take the shape of a war helmet (Fig. 1B), although the external morphology can vary extensively between species [2, 23, 24]. Almost uniformly, the pilidium larva has a body formed by a more or less spherical large episphere, two lateral lappets, and anterior and posterior lobes. The lappets and lobes are used for generating a feeding current and catching particles of food [25]. The mouth opens between the two lateral lobes, and connects to a blind gut through a small vestibule (Fig. 1B). On the opposite pole of the larva—aboral pole—there is an apical tuft (Fig. 1B). The occurrence of this type of larva in many different species suggests that a pelagic planktotrophic pilidium larva was present in the last common ancestor of the Pilidiophora [2, 26] (Fig. 1A). During its lifespan, the typical pilidium larva develops three pairs of rudiments—imaginal discs—as invaginations of the larval epidermis inside the blastocoel. The cephalic pair of imaginal discs forms first [22], from the first quartet micromeres [14], and originates the head of the juvenile. The second pair—trunk discs—and third pair—cerebral organ discs—form subsequently, depending on the feeding of the pilidium [22]. In addition to these three pairs of imaginal discs, an unpaired anterior rudiment forms the proboscis, and an unpaired dorsal mesenchymal disc contributes to the formation of the trunk [22]. The only connection between the larva and the future juvenile is thus the mouth and blind gut, which are conserved during metamorphosis. The growth, differentiation, and eventual fusion of the imaginal discs form the juvenile. A rapid metamorphosis occurs when the juvenile worm is completely formed inside the larval body, and often involves the ingestion of the larval tissue by the hatching juvenile [22, 27].

Although pilidium larvae were considered the typical example of planktotrophic larvae, recent evidences suggest that lecithotrophy is much more common than previously thought, occurring in at least 16 Pilidiophoran lineages [2, 28–32]. Investigation of the metamorphosis of these lecithotrophic pilidium larvae showed that the juvenile forms from imaginal discs similar to those observed in a typical planktotrophic pilidium, and that the larval body—epidermis, apical organ—is also discarded [30, 31]. However, the most extreme case of deviation from the ancestral planktotrophic pilidium occurs in the species *Lineus ruber* (Müller, 1774) and *Lineus viridis* (Müller, 1774). In these two nemerteans, the embryo develops into an intracapsular larval form, which eventually metamorphoses into the juvenile [29, 33–36]. The so-called Schmidt’s (in *L. ruber*) and Desor’s (in *L. viridis*) larvae lack the buccal lappets and the apical organ, but also forms pairs of imaginal discs and a larval epidermis that is discarded during metamorphosis [29, 37]. Additionally, the Schmidt’s larva of *L. ruber* exhibits adelphophagy, and predates on unfertilized eggs that occupy the same egg capsule during development [38, 39]. Therefore, the Pilidiophora emerges as an ideal group to study the developmental implications associated with the evolution of an indirect life cycle and the transition between alternative nutrition modes (planktotrophy, lecithotrophy and adelphophagy) and dispersal strategies (pelagic larvae versus intracapsular larvae). In this context, most of the recent work has focused on understanding the embryonic development of pelagic pilidium larvae [22, 40, 41], whereas little attention has been paid to those lineages exhibiting the most extreme cases of larval adaptation, such as *L. ruber* and *L. viridis*.

In this study, we use molecular approaches to characterize the development and metamorphosis of the adelphophagic intracapsular Schmidt’s larva of the nemertean *L. ruber*. By means of confocal laser scanning microscopy and F-actin staining, we show the formation of the imaginal discs and organ rudiments of the Schmidt’s larva and their subsequent transformation into the definitive tissues of the juvenile. In addition, we analyze the patterns of cell proliferation during Schmidt’s larva formation and metamorphosis. We complement our morphological analyses with the expression patterns of anterior/head markers (*foxQ2*, *six3/6*, *goosecoid*, *orthodenticle*), endomesodermal genes (*foxA*, *GATA456*-*a*, *twist*-*a*), and posterior markers (*even*-*skipped*, *caudal*) to better understand the establishment of the positional and cellular identities in the Schmidt’s larva and early juvenile of *L. ruber*. Our study sheds light into the developmental changes occurred during the evolution of an intracapsular larva from a pelagic form in the group Nemertea, and thus helps to uncover the embryonic and molecular mechanisms related to the diversification of life cycles among metazoans.

## Methods

### Animal collection and embryo care

Gravid specimens of *L. ruber* were found under stones in the coast near Bergen (Fanafjorden; GPS coordinates: 60.251845 north, 5.320947 east) during day low tide in March and April 2014. Animals were kept in aquariums with constantly aerated filtered seawater (FSW) at 14 °C, under a day–night cycle of 13 h of light and 11 h of darkness, and fed once a week with adult *Platynereis dumerilii* (Additional file 1: Video S1). Under these conditions, gravid adults laid egg masses spontaneously over the course of several weeks. Egg masses were collected daily and kept in separate petri dishes at 14 °C, with changes of FSW every other day.

### Embryo fixation

At the desired developmental stage, egg masses were dissected under the stereomicroscope with the aid of a pair of tungsten needles to release the embryos from the egg capsules. Embryos were then transferred to a new petri dish with FSW, relaxed for 15 min in 7.4 % MgCl_2_ (from 20-day-old embryos onwards), and subsequently fixed in 4 % formaldehyde in FSW for 1 h at room temperature (RT). Fixative was removed with 3 washes of 5 min in phosphate buffer saline (PBS) with 0.1 % Tween-20 (PTw). Specimens for immunohistochemistry were washed once more in PTw and stored in 0.1 % sodium azide in PTw at 4 °C. Specimens for whole mount in situ hybridization were washed in 50 % methanol in PTw for 5 min, and dehydrated in 100 % methanol twice for 5 min before storage in pure methanol at −20 °C.

### Proliferation studies

To label proliferative cells at the S-phase of the cell cycle, embryos at particular points of development were dissected from the egg masses and incubated in FSW supplemented with 100 μM of the thymidine analog EdU for 1 h at 14 °C. After incubation, embryos were immediately rinsed in FSW, relaxed in 7.4 % MgCl_2_ (from 20-day-old embryos onwards), and fixed in 4 % formaldehyde in FSW for 1 h at RT. The fixative was removed with 3 washes of 5 min in PTw and samples were stored in 0.1 % sodium azide in PTw at 4 °C. Fluorescent labeling of the incorporated EdU was performed as recommended by Click-it EdU Alexa Fluor 594 imaging kit (Life Technologies, NY, USA), and nuclei were counterstained in a 1:10,000 dilution (v:v) of Sytox Green in PTw.

### Phalloidin labeling

Specimens fixed and stored for immunohistochemistry were washed 3 times in PBS to remove the sodium azide. Actin filaments and nuclei were labeled with 5 U/mL of Alexa 647 phalloidin (Life Technologies, NY, USA) and 1:10,000 Sytox Green in PBT (PBS, 0.2 % TritonX-100, 0.1 % bovine serum albumin) for 1 h at RT. Stained embryos were subsequently washed in PBS for 1 h, and mounted for confocal laser scanning observation (see below).

### Gene expression studies

A fragment of *foxA* and *foxQ2*, and the full-length sequences of *cdx*, *evx*, *GATA456*-*a*, *gsc*, *otx*, *six3/6* and *twist*-*a* [GenBank: KT335961–KT335969] were identified from RNAseq data of mixed developmental stages. Protein alignments were constructed with MAFFT v.7 [42] and poorly aligned regions were removed with Gblocks v.0.91b [43]. RAxML v.8 [44] was used to infer gene orthologies (Additional file 2: Figure S1). Resulting trees were formatted with FigTree and Illustrator CS6 (Adobe). Single colorimetric whole mount in situ hybridization was performed as described elsewhere [45], with the only modification of permeabilizing the samples with proteinase K (10 μg/mL in PTw) for 8 min at RT without shaking. After the whole mount in situ hybridization protocol, embryos were cleared and stored in 70 % glycerol in PTw with a 1:5000 dilution of the nuclear marker DAPI.

### Imaging

EdU-labeled and phalloidin-stained embryos were dehydrated in a graded isopropanol series (75, 85, 95 % in miliQ water, and twice in 100 % isopropanol for 30–60 s each step) and cleared in Murray’s reagent (benzyl benzoate to benzyl alcohol, 2:1, v:v). Cleared samples were imaged under a Leica SP5 confocal laser-scanning microscope (Leica, Wetzlar, Germany). Specimens exhibiting representative expression patterns of the analyzed genes cleared in 70 % glycerol were imaged with an Axiocam HRc connected to an Axioscope Ax10 (Zeiss, Oberkochen, Germany), using bright field Nomarski optics. Images were analyzed with Fiji and Photoshop CS6 (Adobe), and figure plates made with Illustrator CS6 (Adobe). Brightness/contrast and color balance adjustments were applied to the whole image, not parts.

## Results

### Oviposition and timing of development of *Lineus ruber*

**Fig. 2:**
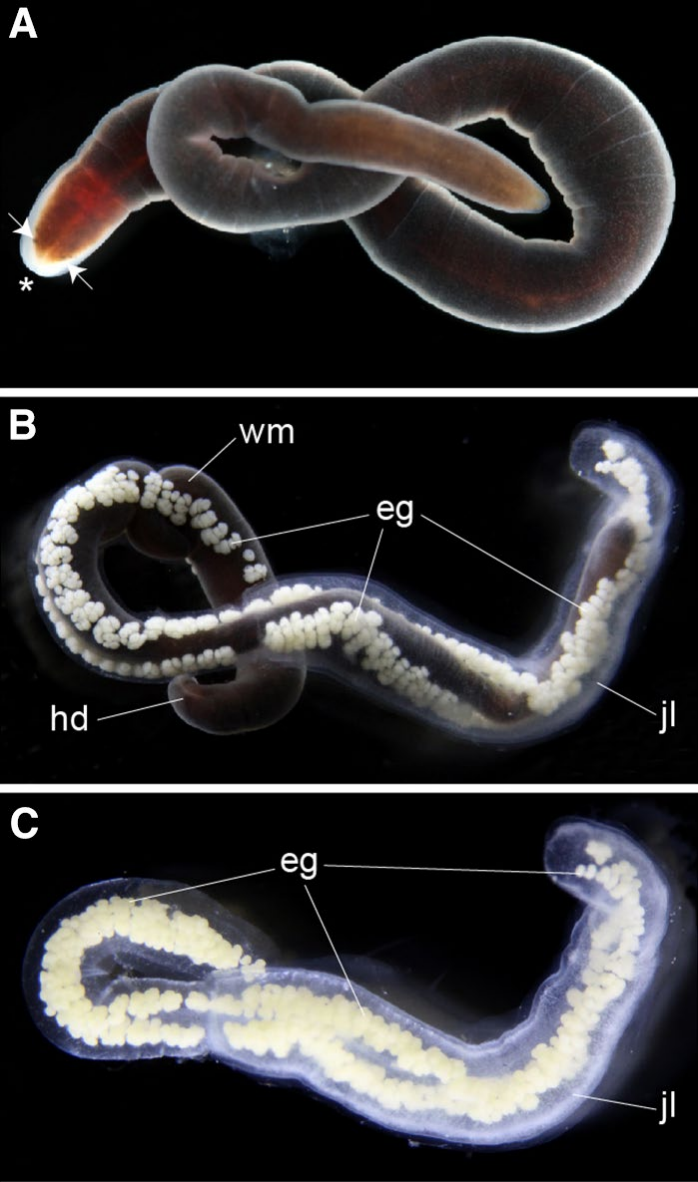
Oviposition in *Lineus ruber*. **A**–**C** Photographs of live specimens taken under the stereomicroscope. **A** Adult specimens of *L. ruber* show the typical elongated ribbon-like appearance of nemertean worms, with a *dark red* pigmentation throughout the body. The head region is of *lighter red color*, and contains several pairs of eyespots (*white arrows*) and a terminal anterior proboscis (*asterisk*). **B** Oviposition occurs through the multiple gonopores located on both lateral sides of the female. Several fertilized oocytes are packed within individual pyriform capsules, which are bounded together inside a jelly mass. As the female releases the eggs and secretes the jelly, it glides out of the forming egg string. **C** Laid egg mass containing multiple egg capsules. The entire development occurs inside this mass. E.g., egg capsules, *hd* head, *jl* jelly cover, *wm* worm

Adult specimens of *L. ruber* collected from the field displayed a dark red pigmentation, which was lighter at the region of the head (Fig. 2A). *L. ruber* is a dioecious species. In mature female animals, multiple individual ovaries are distributed along two lateral rows, with each ovary connecting to the epidermis through a small gonoduct and a gonopore [1]. We did not directly observe fertilization, but it occurs internally after pseudocopulation [46]. Oviposition occurs spontaneously (Fig. 2B). Eggs are released through the lateral gonopores packed into multiple pyriform egg capsules (Additional file 3: Video S2). As the animal releases the eggs, epithelial glands secrete an enclosing jelly. The female nemertean progressively glides out of the jelly and lays the gelatinous cocoon, where development takes place (Fig. 2C). Animals kept under lab conditions over a whole year were still able to reproduce, which indicates that *L. ruber* can display iteroparous reproduction, at least in captivity.

**Fig. 3:**
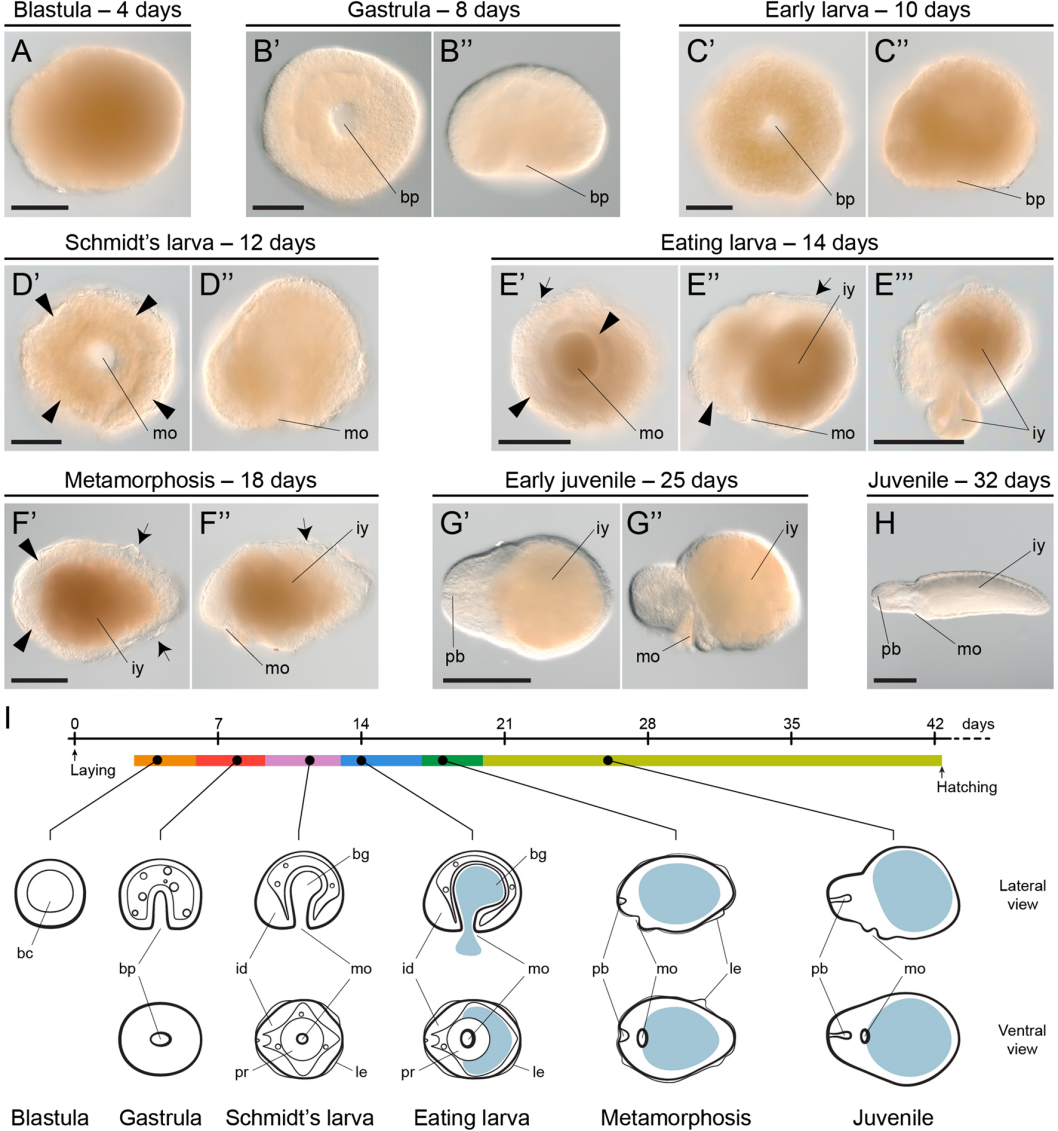
The embryonic development of *Lineus ruber*. **A**–**H** Photographs of fixed dissected embryos of *L. ruber* at representative stages of development. **A** A stereotypical quartet spiral cleavage results in the formation of a yolky coeloblastula. **B′**, **B″** Gastrulation occurs at one pole of the embryo and results in the formation of a central round blastopore and an internal archenteron. **C′**, **C″** The late gastrula shows the first signs of bilateral symmetry, with the future anterior end pointier than the posterior side, which is more round (more evident in **C″**). **D′**, **D″** The Schmidt’s larva forms after 12 days of development. It exhibits an anterior pair and a posterior pair of ectodermal imaginal discs (*arrowheads*), and the blastopore has become the mouth of the larva. A *thick* ectodermal tissue lines the mouth opening. **E′**–**E′′′** The Schmidt’s larva of *L. ruber* can feed on other siblings that occupy the same egg capsule. The ingestion of this yolk material increases the size of the larva and fills up the blind gut. At this stage, the imaginal discs are still visible (*arrowheads*) and a conspicuous epidermis covers the larva (*arrows*). **F′**, **F″** About 18 days after oviposition, the Schmidt’s larva metamorphoses into the juvenile. The imaginal discs (*arrowheads*) expand and the larval epidermis (*arrows*) detaches from the body. **G′**, **G″** After metamorphosis, the juvenile shows a* worm-like* shape, elongated along the anteroposterior axis but not along the dorsoventral axis, mostly because of the high amounts of yolk present in the blind gut. The head region is more developed and the rudiment of the proboscis is now evident. **H** 8 weeks after oviposition, the juveniles are morphologically similar to the adults, and have absorbed part of the ingested yolk. **I** Schematic summary of the embryonic development of *L. ruber* at 14 °C. The* light blue* area depicts the ingested yolk. Drawings are not to scale. **A**, **B″**, **C″**, **D″**, **E″**, **E′′′**, **F″**, **G″**, **H** show lateral views. **B′**, **C′**, **D′**, **E′**, **F′**, **G′** show blastoporal/ventral views. In **C′**–**H** anterior is to the *left*. *bc* blastocoel, *bg* blind gut, *bp* blastopore, *id* imaginal discs, *iy* ingested yolk, *le* larval epidermis, *mo* mouth, *pb* proboscis, *pr* pharyngeal rudiment. *Scale bars* (**A**–**D″**) 50 μm; (**E′**, **E′′′**, **F′**) 100 μm; (**G′**, **H**) 500 μm

Development takes approximately 42 days at 14 °C (Fig. 3) (Additional file 4: Figure S2). Oocytes are rich in yolk content and display a stereotypical spiral cleavage (personal observation and [33]) that results in the formation of a blastula 4 days after oviposition (Fig. 3A) (Additional file 4: Figure S2B). Many embryos display abnormal cell division patterns during cleavage and get arrested at this early stage of embryogenesis. The number of unviable embryos varies between capsules, as well as between egg masses. Gastrulation starts after 6 days of development and occurs by internalization of cells at one pole of the embryo, resulting in the formation of an archenteron and a rounded blastopore (Fig. 3B′, B″) (Additional file 4: Figure S2C). After 10 days of development, the embryo adopts the first signs of bilateral symmetry (Fig. 3C′, C″), with the future anterior end more elongated than the prospective posterior side. The blastopore is still clearly open in a centered position, and the ectodermal walls of the embryo appear more compacted. The intracapsular Schmidt’s larva forms 12 days after oviposition (Fig. 3D′, D″) (Additional file 4: Figure S2D). The larva exhibits a clear bilateral symmetry, with the anterior side pointier than the posterior. The blastopore turns into the mouth of the larva, and the blastoporal ectodermal rim cells appear more developed. In addition, four ectodermal discs occupy the anterior left, anterior right, posterior left and posterior right sides of the larva. A thin ciliated epidermis connects these four discs and encloses the larval body. Internally, the archenteron forms a blind gut. When dissected out of the egg capsules, the Schmidt’s larvae spin and swim slightly. Inside the capsules, they feed on the unviable arrested embryos with which they often cohabitate. As a result of the ingestion of other siblings, the larva increases in size and the blind gut becomes filled with nutrients (Fig. 3E′–E′′′) (Additional file 4: Figure S2E). Approximately 18 days after deposition, the ectodermal discs of the Schmidt’s larva are more developed and extend over the whole body, which is now more elongated along the anteroposterior axis and shows a more worm-like shape (Fig. 3F′, F″). At this point of development, the original larval epidermis covers the body, but starts to detach from the developing definitive epidermis in some regions of the larva. The mouth is still open in an antero-ventral position.

The metamorphosis into the early juvenile is accomplished in most of the larvae about 20 days after oviposition (Fig. 3G′, G″) (Additional file 4: Figure S2G). The early juvenile lacks the larval epidermis. We could not directly follow the fate of this tissue, and it is thus uncertain whether the larval epidermis is ingested by the developing juvenile, as in the sister species *L. viridis* [37], resorbed by the definitive epidermis, or simply discarded. The early juvenile of *L. ruber* has a clear worm-shape and the larval imaginal discs are no longer obvious. The head has an anterior terminal proboscis, but the eyes are not yet formed. The mouth occupies an antero-ventral position and the gut in most of the juveniles is full of yolk content and still blind. Juveniles actively move inside the capsules (Additional file 5: Video S3). As development proceeds, the definitive tissues and organs mature, the yolk is gradually absorbed and the juvenile progressively adopts the morphology of a small adult (Fig. 3H) (Additional file 4: Figure S2H). Juveniles can escape out of the capsule, and glide within the jelly enclosing the egg mass. About 30 days after oviposition, the juveniles show the first signs of eyespots (Additional file 4: Figure S2I), and after about 40 days, they also show body pigmentation (Additional file 4: Figure S2I). Hatching of the juveniles varies between egg masses, but it often occurs after about 40 days of development (Additional file 4: Figure S2I; Additional file 6: Video S4). At the moment of hatching, the size and morphology of the juveniles can vary (Additional file 7: Video S5), but most of them exhibit a normal behavior, being capable of preying on eggs of the annelid *P. dumerilii* (Additional file 8: Video S6). A graphical summary of the main developmental events during *L. ruber* embryogenesis is shown in Fig. 3I.

### The formation of the intracapsular Schmidt’s larva

**Fig. 4:**
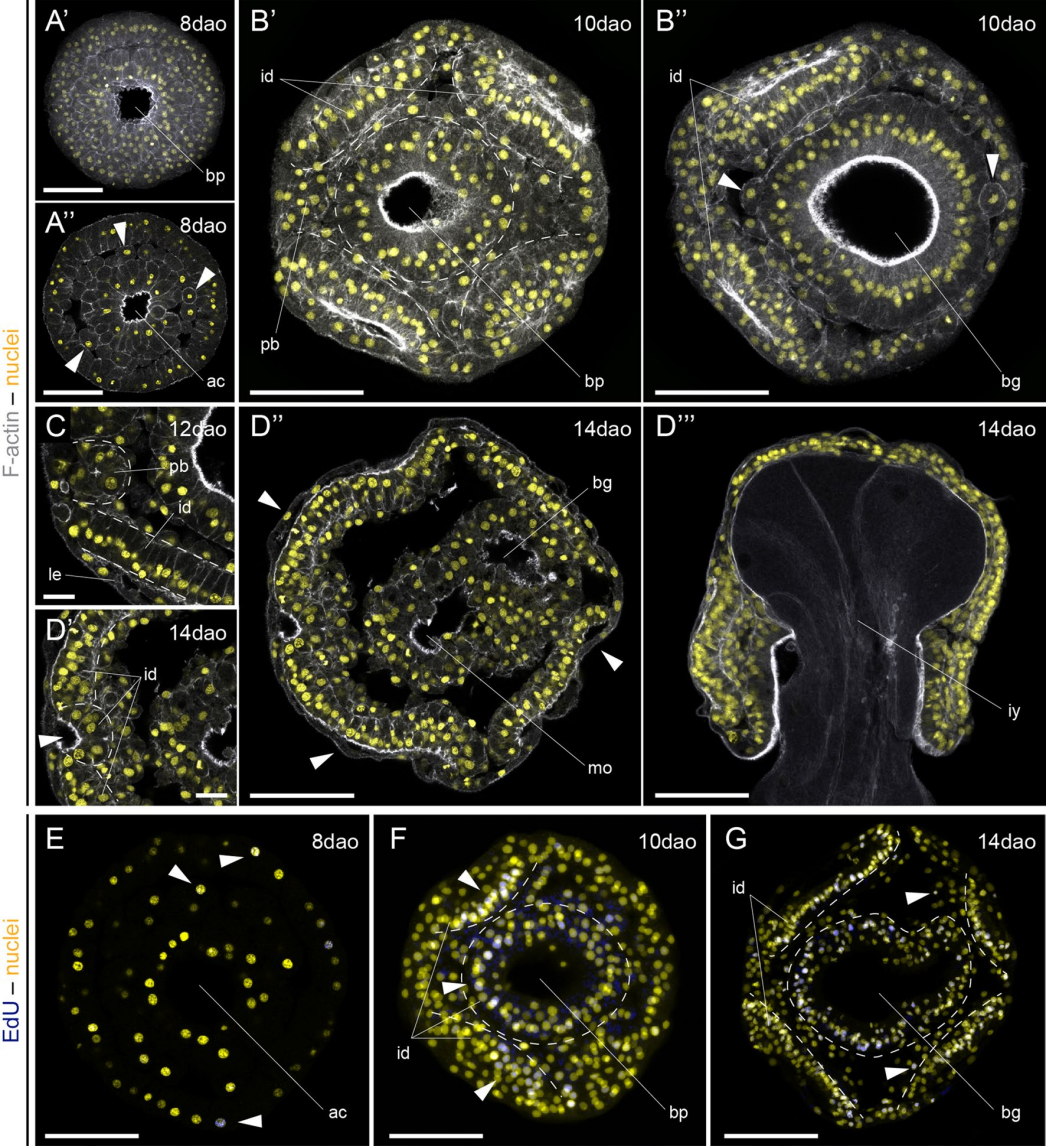
Formation of the Schmidt’s larva in *Lineus ruber*. **A′**–**G** z projections of confocal scans of embryos and larvae labeled against F-actin (*gray* **A′**-**D′′′**) and incorporated EdU (*blue* **E**–**G**) counterstained with the nuclear marker Sytox Green (*yellow*) at 8, 10, 12 and 14 days after oviposition (*dao*). **A′**, **A′′** The gastrula has a central ventral blastopore and a wide open archenteron, with isolated cells occupying the blastocoel (*arrowheads* **A′′**). **B′**–**B′′** After 10 days of development, the embryo shows 4 paired ectodermal discs (marked by *dashed lines* **B′**), an incipient proboscis rudiment, and the ventral ectodermal ring around the blastopore (*dashed circle* **B′**). Internally, the archenteron widens and bends backwards, forming a blind gut. There are still numerous cells in the blastocoel (*arrowheads*). **C** After 12 days, an anterior indentation forms between the two anterior imaginal discs, and the whole embryo becomes covered by a thin epidermis. **D′**–**D′′′** The mature Schmidt’s larva shows 7 separate imaginal discs covered by a larval epidermis (*arrowheads* **D′′**): the anterior terminal proboscis imaginal disc (*arrowhead* **D′**); the four lateral imaginal discs (cephalic pair and trunk pair); the imaginal disc associated with the mouth; and the imaginal disc of the gut. At this stage of development, the larva feeds on other siblings (**D′′′**). **E**–**G** After gastrulation, proliferative cells are scattered and distributed in the ectoderm and cells in the blastocoel (**E**). With the formation of the Schmidt’s larva, proliferation concentrates mostly on the regions that form the imaginal discs (*arrowheads* **F**; imaginal discs indicated by *dashed lines*). However, isolated cells in the space between the imaginal discs also proliferate (*arrowheads* **G**). **A′**–**D′′**, **E**–**G** are ventral views. **D′′′** Is a lateral view, with the dorsal side to the *top*. In all *panels*, anterior is to the *left*. *ac* archenteron, *bg* blind gut, *bp* blastopore, *id* imaginal disc, *iy* ingested yolk, *le* larval epidermis, *mo* mouth, *pb* proboscis. *Scale bars* (**A′**–**B′′**, **D′′**–**G**) 50 μm; (**C**, **D′**) 10 μm

**Fig. 5:**
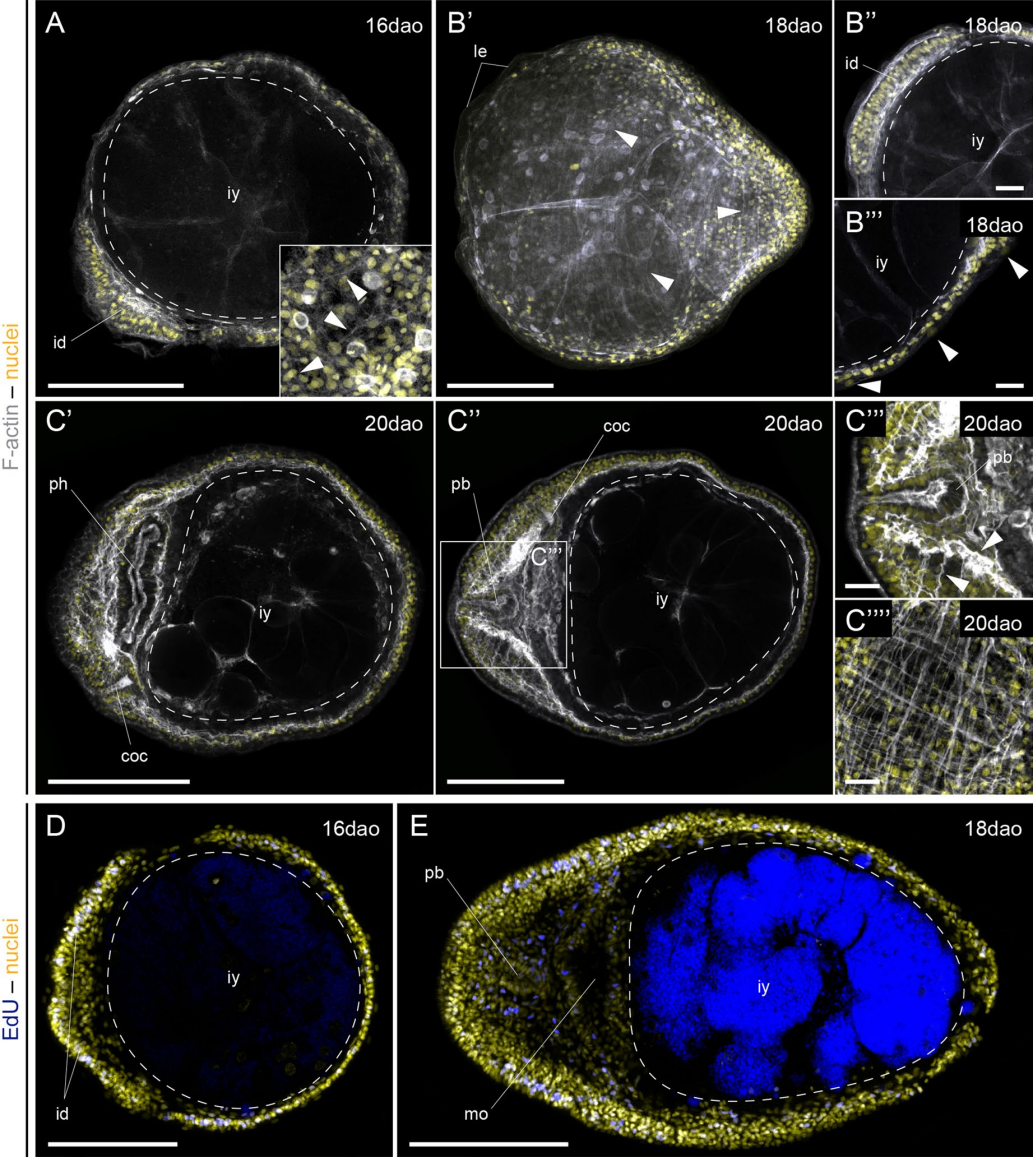
Metamorphosis of the Schmidt’s larva of *Lineus ruber*. **A**–**E** z projections of confocal scans of larvae and early juvenile labeled against F-actin (*gray* **A**–**C′′′′**) and incorporated EdU (*blue* **D**, **E**) counterstained with the nuclear marker Sytox Green (*yellow*) at 16, 18 and 20 days after oviposition (*dao*). **A** Larvae at 16 days of development are rounded and show the first signs of cell differentiation (*arrowheads* in the inset mark F-actin projections). **B′**–**B′′′** After 18 days of development, the larvae are more elongated along the anteroposterior axis, and circular and longitudinal fibers extend below the epidermis (*arrowheads* **B′**). The imaginal discs are more developed (**B′′**) and the larval epidermis is only present in some parts (*arrowheads* **B′′′**). **C′**–**C′′′′** After 20 days of development, the imaginal discs have formed the basic anatomical features of the juvenile. The rhynchocoel occupies the central region of the head, and the mouth opening occupies the antero-ventral side of the animal. The musculature is now much more developed, in particular in the head region around the developing brain lobes (*arrowheads* **C′′′**) and in the body wall (**C′′′′**). **D**, **E** During metamorphosis of the Schmidt’s larva into the juvenile, proliferation is widespread, although more concentrated in the anterior imaginal discs at early stages (**D**) and in the lateral sides of the head in the early juvenile (**E**). **A** is a lateral view, with the dorsal side to the *top*. The rest of the panels are ventral views. In all panels, anterior is to the *left*. In **D**, **E** the *blue* staining in the ingested yolk is background. *coc* cerebral organ canals, *id* imaginal disc, *iy* ingested yolk, *le* larval epidermis, *mo* mouth, *ph* pharynx, *pb* proboscis. *Scale bars* (**A**, **B′**, **C′**, **C′′**, **D**, **E**) 100 μm; (**B′′**, **B′′′**, **C′′′**, **C′′′′**) 25 μm

**Fig. 6:**
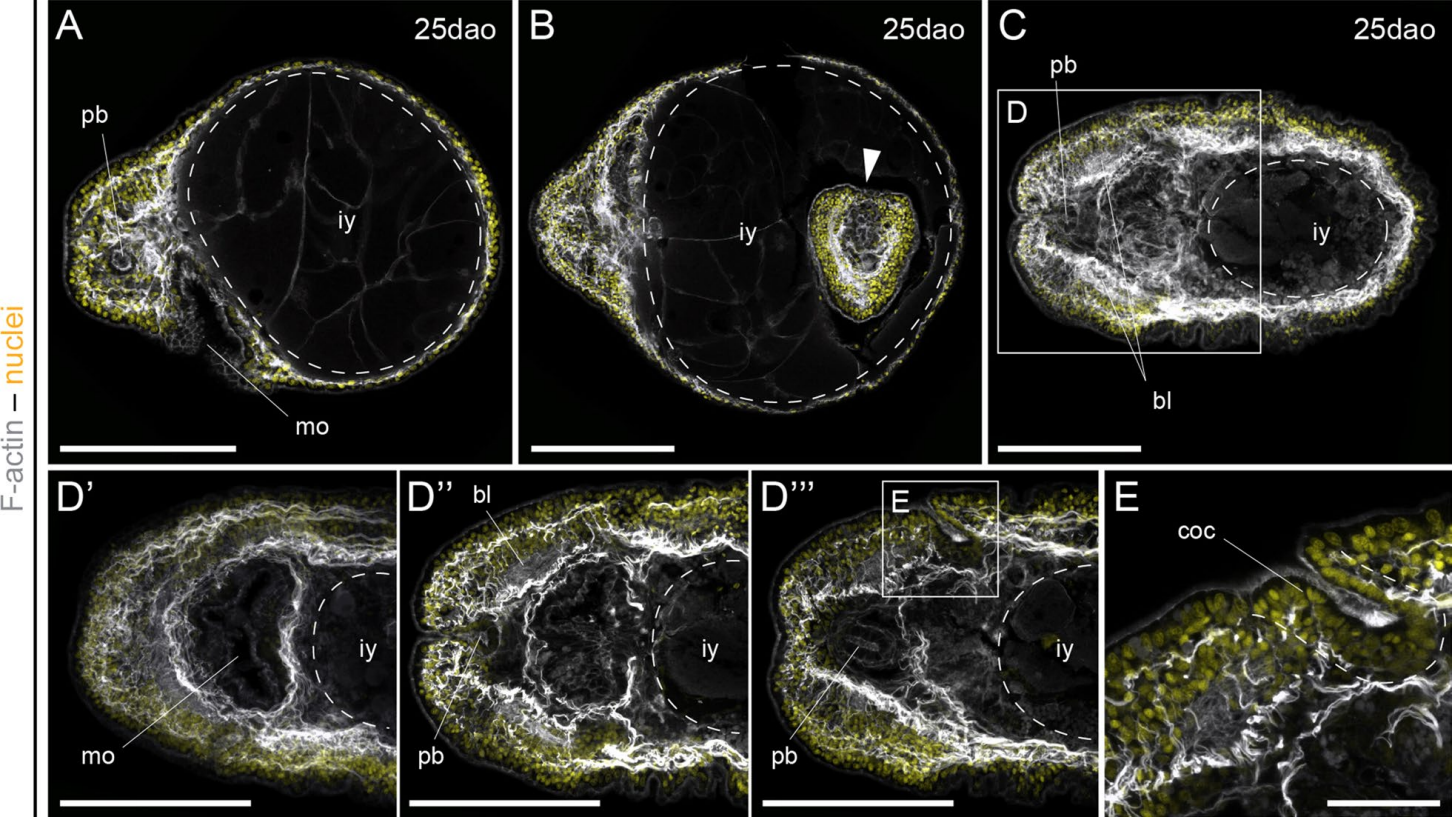
The early juvenile of *Lineus ruber*. **A**–**E** z projections of confocal scans of juveniles labeled against F-actin (*gray*) counterstained with the nuclear marker Sytox Green (*yellow*) 25 days after oviposition (*dao*). **A** Juveniles with a large volume of ingested yolk keep a spherical shape, although they exhibit mature tissues. **B** The adelphophagy of the Schmidt’s larva not only affects unviable embryos, but also developing siblings. **C**–**E** Juveniles with a moderate quantity of yolk soon adopt a worm-like shape. The body wall musculature is well developed and the head region has a muscular mouth (**D′**), and a central rhynchocoel with the two brain lobes at each side (**D′′**–**D′′′**). Posterior to the brain, the ectoderm on the left and right sides of the head invaginates and forms the cerebral organ canal (**D′′′**, **E**), the connection of the cephalic organ with the exterior. Note in (**E**) the particular ciliation of the inner canal compared with the outer epidermis. **A** A lateral view, with the dorsal side to the *top*. **B**–**E** Are ventral views. In all *panels*, anterior is to the *left*. *bl* brain lobes, *coc* cerebral organ canal, *iy* ingested yolk, *le* larval epidermis, *mo* mouth, *pb* proboscis. *Scale bars* (**A**–**D′′′**) 100 μm; (**E**) 25 μm

To better characterize the development of the Schmidt’s larva, we first analyzed the patterns of actin labeling from gastrula to early juvenile stages by confocal laser scanning microscopy (Figs. 4, 5, 6). As also observed under transmitted light (Fig. 3B′), the 8-day-old gastrula shows a central blastoporal opening in the future ventral side of the animal that connects with an internal archenteron (Fig. 4A′, A″). Internally, the archenteron cavity and the cells lining it occupy most of the blastocoel, which is also populated by isolated cells (Fig. 4A″). We could not discriminate whether these come from the endoderm or the ectoderm.

After 10 days of development, the embryo shows the cephalic (anteriorly) and the trunk (posteriorly) pairs of discoidal ectodermal concentrations, clearly segregated from the ring of ectodermal cells that surround the blastoporal opening (Fig. 4B′). In addition, the first signs of the unpaired proboscis rudiment are visible (Fig. 4B′). The archenteron cavity is expanded and bent backwards (Fig. 4B′′), and isolated cells are still present inside the former blastocoel. After 12 days, the embryo adopts the appearance of a Schmidt’s larva (Fig. 3D′, D′′). At this stage, the ectodermal discs are monostratified epitheliums (Fig. 4C), and the proboscis rudiment is more evident. Importantly, a thin ciliated epidermis now covers the entire surface of the larva (Fig. 4C). After 14 days of development, the indentation of the proboscis rudiment is more pronounced (Fig. 4D′). The Schmidt’s larva is now composed of at least seven distinct epithelial aggregates that will be the source of the definitive tissues of the juvenile during metamorphosis: an unpaired anterior proboscis rudiment, two cephalic discs, the ventral mouth/pharynx rudiment, the internal endodermal blind gut, and two trunk discs (Fig. 4D′′). We could not identify a separate pair of cerebral organ discs at this stage (Additional file 9: Figure S3A), which is present in most of the pilidium larvae [17] and was previously described in the Schmidt’s larva [35]. Likewise, we did not observe the formation of a well-defined dorsal rudiment (Additional file 9: Figure S3B). At this stage, the Schmidt’s larva feeds on other siblings, filling the blind gut with yolk (Fig. 4D′′′). Finally, the analysis of EdU incorporation during the formation of the Schmidt’s larva shows that cell proliferation is mostly concentrated in the regions of the embryo where the imaginal discs form (Fig. 4E–G), although isolated cells in the internal cavity also proliferate, which is similar to what is observed in pelagic planktotrophic pilidium [47].

### Metamorphosis of the Schmidt’s larva and organogenesis in the early juvenile

After 16 days of development, the Schmidt’s larva has significantly increased in size due to the feeding event, the blind gut is expanded and the blastocoel obliterated (Fig. 5A). The larva, however, still has a spherical morphology. At this stage, cells below the surface epidermis start to form actin-positive projections (inset in Fig. 5A), which might be the earliest signs of muscle development. Eighteen days after oviposition, the larva adopts a worm-like morphology, with the posterior tip of the trunk more elongated (Fig. 5B′). The presence of actin-positive fibers below the epidermis is more evident, and the cephalic discs are much more developed (Fig. 5B′′). The larval epidermis is still present in some regions of the body (Fig. 5B′′′). After 20 days of development, the larva of *L. ruber* has fully metamorphosed into a juvenile (Fig. 5C′, C′′). At this stage, the mouth has a clear antero-ventral position, with a well-developed musculature (Fig. 5C′). The head shows two bilateral lobes, with a conspicuous longitudinal and transverse musculature, and the cerebral organ canals become visible (Fig. 5C′′). In addition, longitudinal and circular muscle fibers form the body wall musculature of the developing juvenile (Fig. 5C′′′′). During metamorphosis, the amount of proliferative cells increases (Fig. 5D, E). After 16 days of development, they are abundant in the cephalic discs, although an important number of proliferative cells are also scattered throughout the trunk region. In the early juvenile, proliferation occurs throughout the entire body, but most intensely on the lateral sides of the head.

After 25 days of development, the shape of the juveniles can vary considerably, mostly depending on the amount of yolk ingested during the larval stage (Fig. 6A–C). Those with more yolk retain a more rounded morphology, although they still exhibit a well-developed musculature, proboscis, mouth, pharynx, and head region (Fig. 6A). Some of these large specimens contain another developing embryo (Fig. 6B), which demonstrates that the Schmidt’s larva not only feeds on arrested embryos, but can also ingest apparently viable embryos. Finally, those juveniles with less ingested yolk exhibit a more mature anatomy (Fig. 6C–E). The elongated shape along the anteroposterior axis is more evident, and the body is also flatter along the dorsoventral axis. The body wall musculature is conspicuous (Fig. 6C), and the pharynx shows a strong muscular plexus (Fig. 6D′, D′′). The mouth opens behind the brain, which is composed of two bilateral lobes surrounded by the head musculature (Fig. 6D′′). Some muscular fibers cross the brain lobes (Fig. 6D′′). The anterior median region is occupied by the proboscis (Fig. 6D′′, D′′′), and the dorsal ectoderm posterior to the brain exhibits well formed cerebral organ canals on each side of the head (Fig. 6D′′′, E) [48]. They exhibit a heavily ciliated epidermis and terminate in a wider ampulla, in correspondence with the putative chemotactile function [1]. Labeling against F-actin cannot resolve the presence of a cerebral organ associated with the cephalic slit at this stage. Altogether, these results indicate that the juvenile of *L. ruber* acquires the basic anatomy of the adult after 25 days of development at 14 °C.

### Molecular specification during the formation of the Schmidt’s larva

To characterize in greater detail the formation of the Schmidt’s larva, we identified and studied the expression pattern of genes involved in the specification of anterior and cephalic tissues (*foxQ2*, *six3/6*, *goosecoid* [*gsc*], *orthodenticle* [*otx*]) [40, 45, 49–60], endomesodermal cell fates (*foxA*, *GATA456*-*a*, *twist*-*a* [*twi*-*a*]) [56, 60–70], and posterior territories (*even*-*skipped* [*evx*] and *caudal* [*cdx*]) [59, 60, 70–77] during blastula and gastrula stages, and in the Schmidt’s larva.

**Fig. 7:**
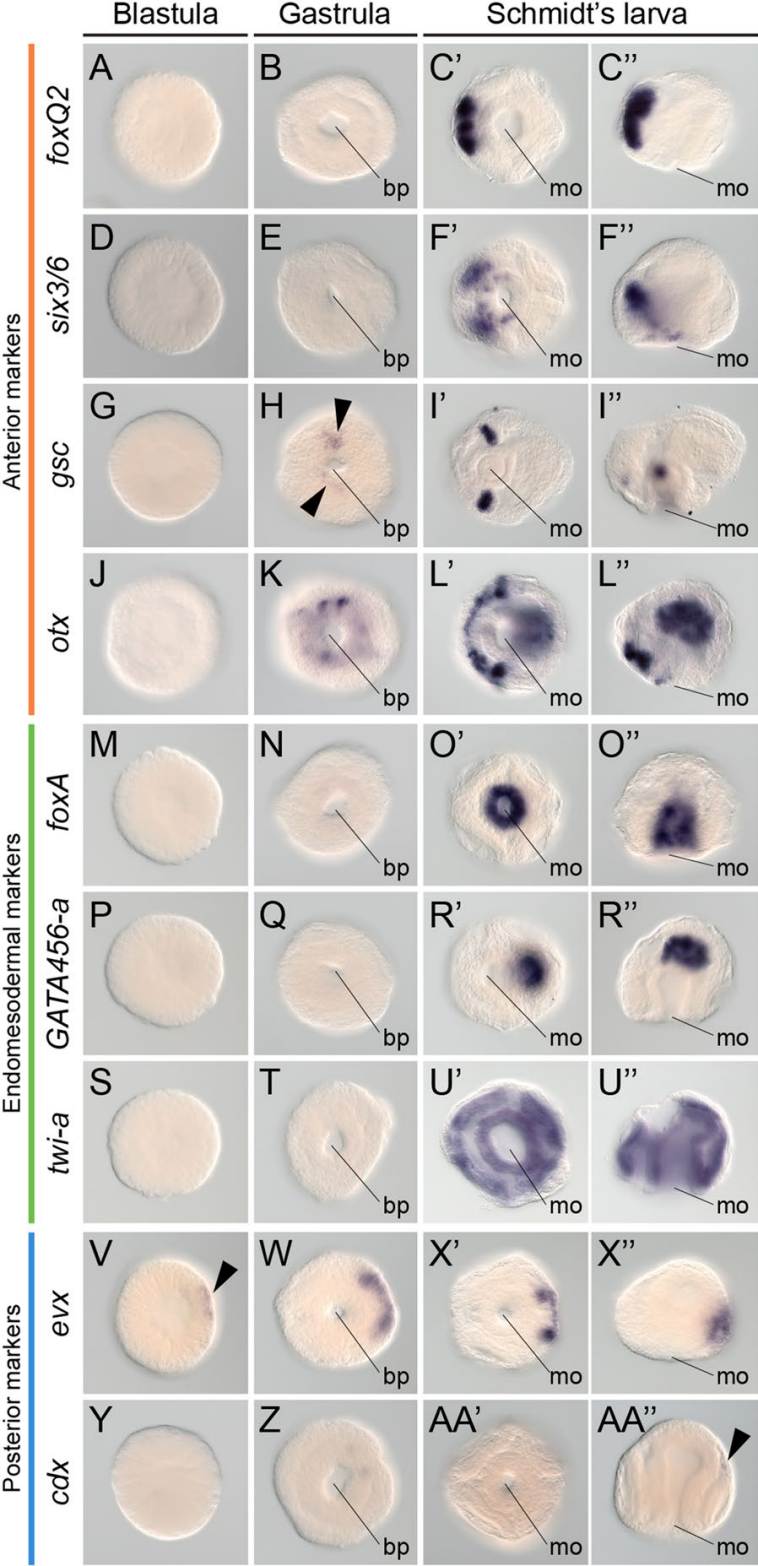
Gene expression during Schmidt’s larva formation in *Lineus ruber*. **A**–**AA′′** Whole-mount in situ hybridization in blastulae, gastrulae and Schmidt’s larvae of *L. ruber*. **A**–**C′′** *foxQ2* is expressed in the anterior region of the Schmidt’s larva. **D**–**F′′** *six3/6* is expressed in the anterior cephalic imaginal discs and in the anterior mouth. **G**–**I′′** *gsc* is detected in two ventro-lateral clusters at each side of the blastopore (*arrowheads* **H**) and mouth. **J**–**L′′** *otx* is expressed around the blastopore opening, and in the anterior imaginal discs and gut of Schmidt’s larva. **M**–**O′′** *foxA* is expressed in the mouth and larval pharynx. **P**–**R′′** *GATA456*-*a* is detected in the blind gut of the larva. **S**–**U′′** *twi*-*a* is expressed in all imaginal discs of the larva. **V**–**X′′** *evx* is expressed in one pole of the blastula (*arrowhead* **V**), one side of the gastrula and in the posterior end of the Schmidt’s larva. **Y**–**AA′′** *cdx* is weakly detected in the posterior end of the larva (*arrowhead* in **AA′′**). All panels of blastula embryos are lateral views, and *panels* of gastrulae are blastoporal views. In the Schmidt’s larva, *panels* on the *left* are ventral views and *panels* on the *right side* are lateral views. In all cases, anterior of the Schmidt’s larva is to the *left*. *bp* blastopore, *mo* mouth

Anterior genes are first detected at the gastrula stage (Fig. 7A–L′′). At this stage, *gsc* is expressed in two clusters of ectodermal cells of the blastoporal rim (Fig. 7H), and *otx* is more broadly expressed around the blastoporal opening (Fig. 7K). All analyzed anterior genes are expressed in the Schmidt’s larva. In the intracapsular larva, *foxQ2* is expressed in the most anterior region of the cephalic discs and the proboscis rudiment (Fig. 7C′, C′′), *six3/6* is expressed in the anterior region of the cephalic discs and the anterior ectoderm of the mouth (Fig. 7F′, F′′), *gsc* is expressed in two antero-lateral clusters of cells (Fig. 7I′, I′′), and *otx* is expressed in the cephalic discs, anterior mouth, and the blind gut (Fig. 7L′, L′′).

Endomesodermal genes are only detected late in the Schmidt’s larva (Fig. 7M–U′′). The fox gene *foxA* is expressed in the mouth and pharynx of the larva (Fig. 7O′, O′′), the endodermal gene *GATA456*-*a* is detected in the blind gut (Fig. 7R′, R′′) and the mesoderm-associated gene *twi*-*a* is broadly expressed in all the imaginal discs of the Schmidt’s larva (Fig. 7U′, U′′).

Finally, the posterior gene *evx* is expressed already at one pole of the blastula (Fig. 7V), and is later restricted to the presumably posterior side of the gastrula (Fig. 7W) and the posterior end of the Schmidt’s larva (Fig. 7X′, X′′). The gene *cdx* is however only weakly detected at the posterior tip of the Schmidt’s larva (Fig. 7Y–AA′′). Altogether, the expression of anterior, endomesodermal and posterior genes suggest that the establishment of the basic molecular regionalization of the embryo of *L. ruber* occurs during the formation of the Schmidt’s larva.

### Molecular specification during metamorphosis and organogenesis in the early juvenile

**Fig. 8:**
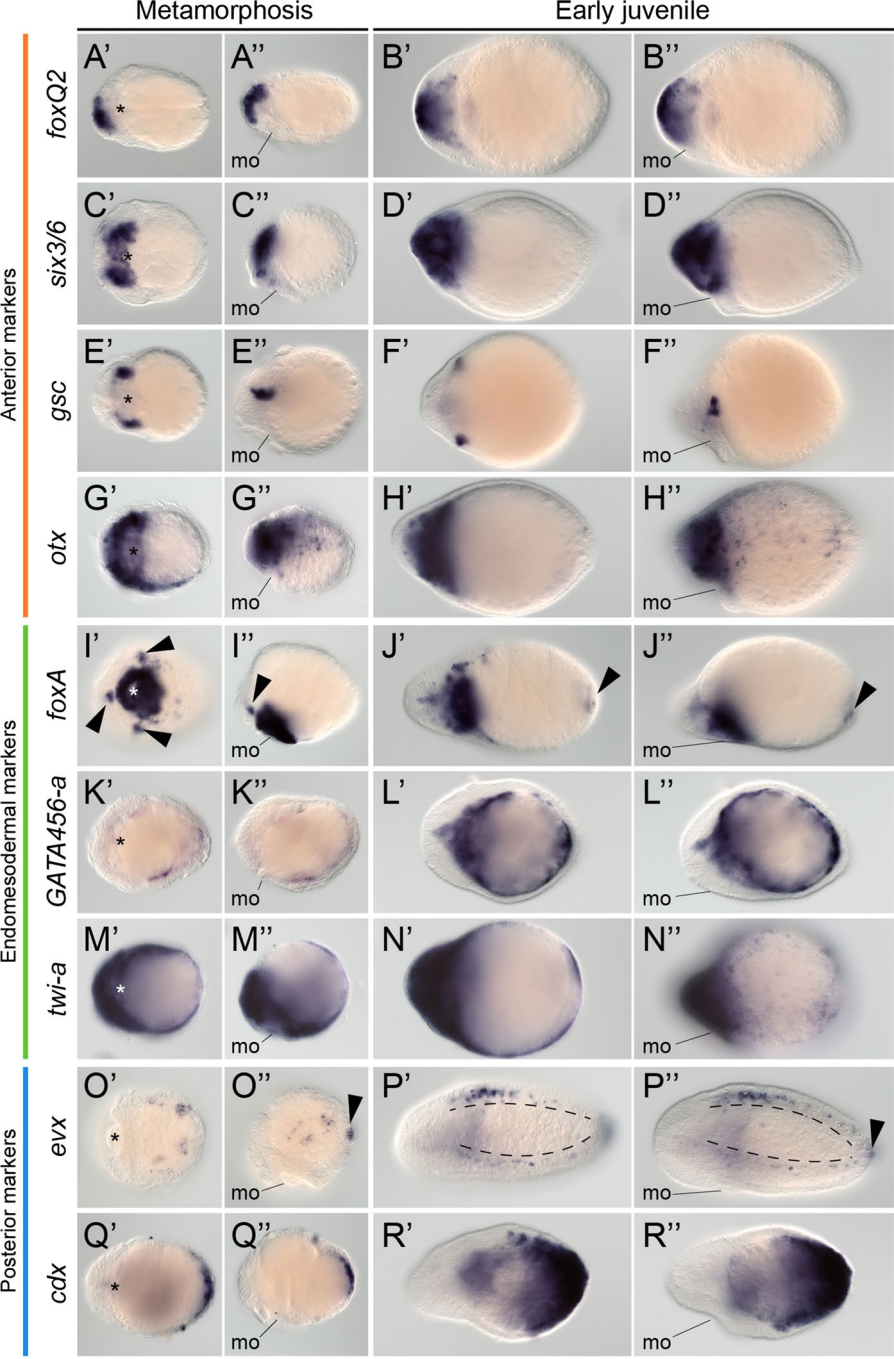
Gene expression during metamorphosis in *Lineus ruber*. **A′**–**R′′** Whole-mount in situ hybridization during metamorphosis and in early juveniles of *L. ruber*. **A′**–**B′′** *foxQ2* is expressed in the anterior end of the metamorphic embryo and juvenile. **C′**–**D′′** *six3/6* is detected in the anterior mouth and cephalic lobes of the metamorphic embryo and in the head region and anterior mouth of the juvenile. **E′**–**F′′** *gsc* is expressed into antero-lateral domains during metamorphosis and in the juvenile. **G′**–**H′′** *otx* is broadly expressed in the anterior region and dorsal side of the metamorphic larva and juvenile. **I′**–**J′′** *foxA* is expressed in the mouth and pharynx, as well as three clusters (*arrowheads* **I′**, **I′′**) of the metamorphic larva. In the juvenile, *foxA* is expressed in the mouth, inner head, ventral side and posterior end of the endoderm (*arrowheads* **J′**, **J′′**). (**K′**–**L′′**) *GATA456*-*a* is detected in the gut during metamorphosis and in the juvenile. **M′**–**N′′** *twi*-*a* is broadly expressed throughout the metamorphic larva and juvenile. **O′**–**P′′** *evx* is detected in the posterior tip of the larva (*arrowhead* **O′′**) and in postero-lateral scattered cells during metamorphosis. In the juvenile, *evx* is expressed in the posterior end of the animal (*arrowhead* **P′′**) and in the lateral and dorsal nerve cords. **Q′**–**R′′** *cdx* is expressed in posterior end of the larva during metamorphosis and in the posterior end and endoderm of the juvenile. In metamorphic larvae and early juveniles, *panels* on the *left* are ventral views and *panels* on the *right side* are lateral views. In all cases, anterior is to the *left*. *mo* mouth

We next studied the expression of the anterior, endomesodermal and posterior markers during metamorphosis and in the early juvenile of *L. ruber*. During metamorphosis, the anterior gene *foxQ2* is expressed in the most anterior region of the cephalic discs and in the proboscis (Fig. 8A′, A′′). In the juvenile, *foxQ2* is expressed in the anterior head, including the proboscis (Fig. 8B′, B′′). The gene *six3/6* is expressed in the cephalic discs and in the anterior mouth during metamorphosis (Fig. 8C′, C′′), and broadly in the head region and anterior mouth in the juvenile (Fig. 8D′, D′′). The gene *gsc* is expressed in two antero-lateral domains during metamorphosis (Fig. 8E′, E′′), and in two clusters of cells associated to the cerebral organ canals and that might correspond to the cerebral organs in the juvenile (Fig. 8F′, F′′) (Additional file 10: Figure S4). Finally, the anterior gene *otx* is expressed broadly during metamorphosis, mostly in the anterior region, the mouth and scattered cells on the dorsal side (Fig. 8G′, G′′). In the early juvenile, *otx* is expressed in the head region, and in subsurface isolated cells of the dorsal side of the trunk (Fig. 8H′, H′′). The cell types expressing *otx* on the dorsal side are unknown.

During metamorphosis, the endomesodermal marker *foxA* is expressed strongly in the mouth, as well as in three clusters of cells anterior and lateral to the mouth and in scattered cells of the ventral region of unknown type (Fig. 8I′, I′′). In the juvenile, *foxA* is detected in the mouth, ventral side of the trunk and in the posterior tip (Fig. 8J′, J′′). The endodermal marker *GATA456*-*a* is expressed in the blind gut during metamorphosis (Fig. 8K′, K′′), and in the definitive endoderm and central part of the head in the juvenile (Fig. 8L′, L′′). The mesodermal gene *twi*-*a* is expressed throughout the entire body during metamorphosis and in the early juvenile (Fig. 8M′–N′′).

The posterior gene *evx* is expressed in a small cluster of cells at the posterior tip during metamorphosis, as well as in two lateral bands of scattered cells in the lateral sides of the larva, presumably the developing lateral nerve cords (Fig. 8O′, O′′). In the early juvenile, expression of *evx* is detected in the lateral nerve cords, the dorsal nerve cord and in the posterior end of the juvenile (Fig. 8P′, P′′). The gene *cdx* is strongly expressed in the posterior end during metamorphosis (Fig. 8Q′, Q′′) and in the posterior ectoderm and endoderm of the juvenile (Fig. 8R′, R′′). Therefore, the expression of anterior, endomesodermal and posterior genes during metamorphosis and in the juvenile together is congruent with the fates of the imaginal discs assigned after the morphological analyses in the Schmidt’s larva of the nemertean *L. ruber*.

## Discussion

### The embryonic development of the Schmidt’s larva

The typical planktotrophic pilidium larva takes the shape of a helmet, with a large hollow episphere originating from the blastocoel [2, 17, 24]. On top of the episphere, there is an apical tuft, and the mouth opens on the opposite pole in between four lobes: one anterior, one posterior, and two long lateral lobes. Likely connected to their active swimming and predatory behavior [25], the pilidium larva has a well-developed neuromuscular system [22, 78, 79]. The modified lecithotrophic pilidium larvae retain some of the basic morphological features of their planktotrophic counterparts, such as the presence of ciliary bands—in some cases—and an apical tuft [28]. However, the oral lobes are missing [2, 30–32], consistently with the absence of a planktotrophic feeding behavior. Although not yet described in lecithotrophic pilidium larvae, the presence of ciliary bands and an apical tuft that exhibit complex behaviors [28] supports that there is likely a neuromuscular system in these larvae. Therefore, in all pelagic pilidium larvae, regardless of their trophic mode, the larval body involves the differentiation of several cell types and larval-specific tissues, in addition to the imaginal discs that will form the juvenile. Our morphological study on *L. ruber* indicates that this is not the case in the intracapsular Schmidt’s larva (Fig. 4). The formation of the Schmidt’s larva is intimately linked to the development of the imaginal discs that will originate the definitive juvenile, and the transitory larval epidermis is the only larval-specific tissue detected [37]. Despite the fact that the Schmidt’s larva of *L. ruber* actively feeds on other siblings and can spin inside the egg capsule and when dissected out, we did not observed muscular fibers or any conspicuous ciliary band. Swimming is thus likely controlled by the normal ciliation of the larval epidermis, and the uptake of food material might be stimulated by the strong ciliation of the pharyngeal tract.

In a planktotrophic pilidium, only the cephalic discs form during embryonic development [14, 22]. The other two pairs—the trunk and the cerebral organ discs—are feeding-dependent, and form days or weeks after the pilidium has entered the water column. In lecithotrophic pilidium larvae, a relationship between feeding and imaginal disc formation is obviously not observed, and all pairs of discs form more or less at the same time, just after gastrulation [30]. This situation is similar to what we observe in the intracapsular Schmidt’s larva of *L. ruber* (Fig. 4). Unlike the typical planktotrophic pilidium, which has three pairs of imaginal discs, we only observe two pairs in the Schmidt’s larva of *L. ruber*, namely a cephalic pair and a trunk pair. Soon after these two pairs are formed, the proboscis rudiment appears. We could not observe an unpaired dorsal disc, although there are scattered mesenchymal cells in that region of the larva that could contribute to the formation of the dorsal side of the juvenile. Previous reports described the presence of a third pair of discs associated with the formation of the cerebral organs [29]. Our morphological analyses cannot resolve the presence of a distinct third pair, but we do observe a specific region of the larva that seems to be committed to the formation of these sensory organs (see below). Our results are, however, consistent with what was described for the lecithotrophic pilidium of *Micrura akkeshiensis* [30]. Although further studies are needed to completely understand the exact cellular mechanisms of imaginal disc formation in the Schmidt’s larva of *L. ruber*, our data suggest that the transition to lecithotrophy is associated with a heterochronic shift—predisplacement—on the growth of the imaginal discs, which form earlier than in planktotrophic forms.

The presence of clearly differentiated transitory larval tissues and the formation of the juvenile from distinct undifferentiated growth zones results in a drastic, rapid metamorphosis in the planktotrophic pilidium [22]. The worm forms inside the hollow episphere of the larva and at some point hatches and devours the remaining larval tissues [22, 27]. Lecithotrophic pilidium metamorphose more or less similarly, with the worm growing inside the larva and eventually hatching and devouring the remaining larval epidermis and apical organ [30, 31, 37]. In the Schmidt’s larva of *L. ruber*, the metamorphosis appears to be more gradual. In support of this observation, cell differentiation, such as the formation of the first myocytes and muscle fibers, starts before the larval epidermis is shed (Fig. 5), and the molecular regionalization of the early Schmidt’s larva corresponds to that exhibited by the early juvenile (Figs. 7, 8; see below). Our results thus indicate that the transition between the Schmidt’s larva and the juvenile, which happens after about 18 days of development, mostly involves the differentiation of the definitive cell types and the establishment of the organ rudiments of the worm (e.g., the brain lobes, the cerebral organs), as well as the loss of the larval epidermis. The early juvenile thus formed still needs several days to adopt the final worm-like shape, and even weeks to exhibit a more mature morphology. This might be related to the intracapsular mode of development, since the juveniles can stay in a closed protected environment for around 20 days after metamorphosis.

### The molecular patterning of the Schmidt’s larva

**Fig. 9:**
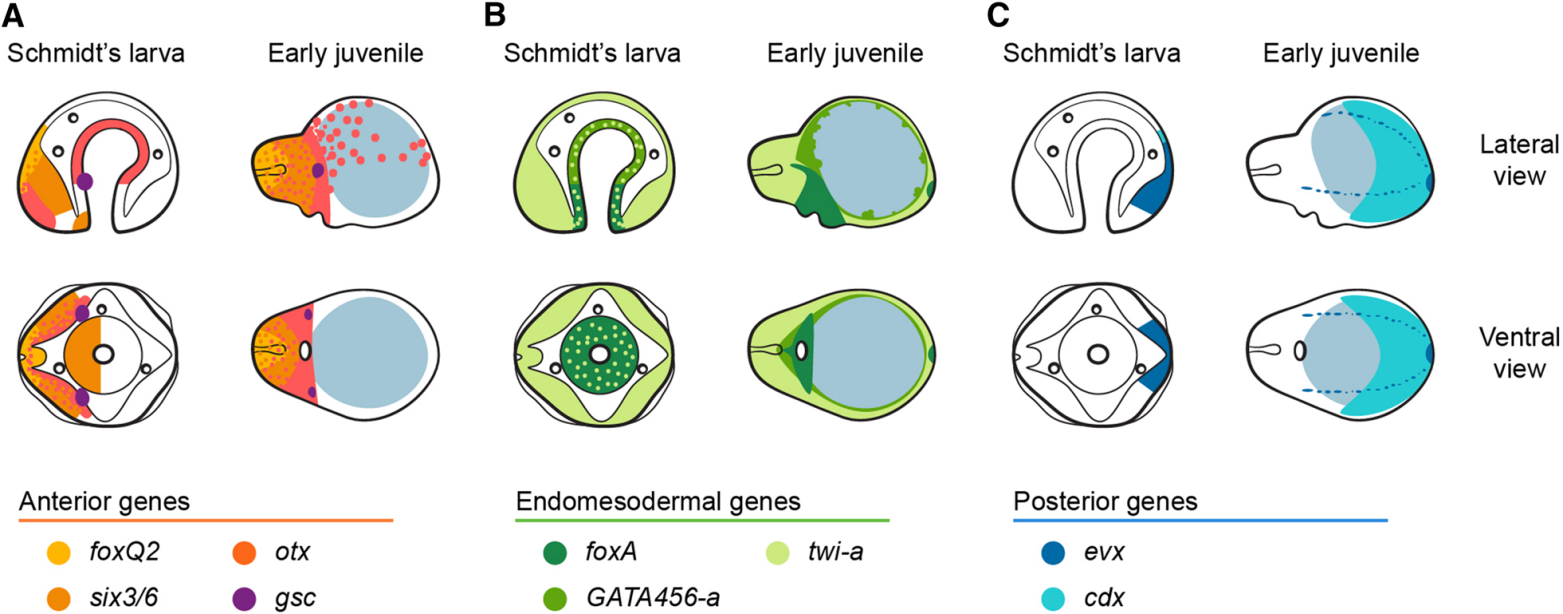
Summary of gene expression during *Lineus ruber* embryonic development. **A**–**C** Schematic representation of the expression patterns of anterior, endomesodermal, and posterior markers, respectively, in the Schmidt’s larva and early juvenile of *L. ruber*. **A** The anterior/head markers *foxQ2*, *six3/6* and *otx* are expressed in the cephalic imaginal discs and head of the juvenile. Additionally, *otx* is expressed in the gut of the Schmidt’s larva and scattered dorsal cells of the juvenile trunk. The gene *gsc* is expressed in two antero-lateral clusters of the Schmidt’s larva and in the cerebral organs of the juvenile. **B** The endodermal genes *foxA* and *GATA456* are complementary expressed in the blind gut of the Schmidt’s larva and early juvenile. The mesodermal gene *twist*-*a* is expressed broadly in all imaginal discs and juvenile tissues. **C** The posterior markers *evx* and *cdx* are expressed in the posterior end of the Schmidt’s larva and juvenile. The depicted expression patterns are for guidance and not necessarily represent exact expression domains. *Dots of color* indicate overlapping expression. The ingested yolk is depicted with a *light blue circle*. Drawings are not to scale

The study of gene expression in different spiralian larvae has revealed important similarities in the molecular underpinnings of cell type specification and embryonic patterning [80, 81]. To improve our understanding of the developmental events leading to the formation of the Schmidt’s larva and its metamorphosis into the juvenile, we analyzed the expression patterns of nine conserved genes associated with the formation of anterior/head structures, the endomesoderm, and posterior embryonic regions (Figs. 7, 8). The genes *foxQ2*, *six3/6* and *otx* are expressed in the most anterior regions of the trochophore larva of annelids and molluscs [51, 52, 57, 82], the trilobed larva of the brachiopod *Terebratalia transversa* [50], and the brain and anterior sensory organs of planarian flatworms [53]. In nemerteans, the gene *six3/6* is expressed in the anterior invaginations of the hoplonemertean larva of *Pantinonemertes californiensis* [59] and in the cephalic discs and apical organ of the pilidium larva of *Micrura alaskensis* [40, 59]. Consistently, we observed expression of *foxQ2*, *six3/6* and *otx* in the most anterior region of the cephalic imaginal discs of the Schmidt’s larva and head of the juvenile (Fig. 9A). Importantly, the gene *gsc*, which is related to the formation of the oral nervous system of many spiralian larvae [51, 56], is expressed in two lateral clusters of cells of the Schmidt’s larva and later on the developing cerebral organs of the juvenile (Fig. 9A). Although we could not track its specific origin, the expression of *gsc* suggests that the cerebral organs develop from a region morphologically different from the other imaginal discs that becomes specified early in development. In this respect, there seems to be variation between pelagic pilidium larvae, since *M. akkeshiensis* seems to form the cerebral organs from pharyngeal invaginations, rather than separate imaginal discs [30].

Cell lineage studies established that the fourth quartet micromeres and macromeres form the endoderm of the planktotrophic pilidium larva, and that the larval mesoderm—musculature—arises from the blastomeres 4d (endomesoderm) and 3a, 3b (ectomesoderm) [13, 14]. Early descriptions of the development of the Schmidt’s larva suggested that the larval mesoderm also arises from a 4d blastomere [33]. Although cell lineage analyses are still lacking, our observations indicate that the blastomeres internalized during gastrulation contribute to the larval gut, but the absence of clearly differentiated muscular cells hamper defining the mesoderm in the Schmidt’s larva. In this respect, the origin of the loose cells occupying the blastocoel is unclear, and could come from cells delaminated from either the ectoderm or the gut epithelium. Additionally, the ubiquitous expression of the mesodermal marker *twist* [63, 65, 66, 69] in all imaginal discs of the Schmidt’s larva (Fig. 9B) suggests that mesodermal differentiation occurs simultaneously throughout the body. Differently from the situation observed with the development of the mesoderm, the expression of the gut-related genes *foxA* and *GATA456*, which have been related to the molecular specification and regionalization of the spiralian gut [62–64], indicates that the blind gut of the Schmidt’s larva and of the early juvenile still retains the ancestral spiralian patterning of an anterior gut region (*foxA* positive) and a mid-portion (*GATA456* positive) (Fig. 9B). We did not observe the formation of a posterior anal opening after 25 days of development. However, the juveniles seem to have a clear through gut at the moment of hatching, suggesting that the anus opens at some point in between these time points.

The paraHox gene *cdx* and the homeotic gene *evx* are expressed in the posterior region of most analyzed spiralian embryos [71, 75, 77]. Consistently, they are expressed in the posterior region of the trunk imaginal discs in the Schmidt’s larva, and in the posterior tip of the early juvenile (Fig. 9C). Strikingly, *evx* is the only gene expressed at the blastula stage, and none of the studied genes, which cover a wide range of axial fates and cell types, were expressed during cleavage stages. However, *six3/6* is expressed at the blastula stage (blastosquare) in *Micrura alaskensis* [40]. Despite this difference, our data and other studies of gene expression during nemertean development [40, 59] contrast with what is reported on other spiral cleaving embryos, in which the early establishment of blastomere lineages is associated with an early expression of lineage-associated genes, including most of the genes reported in our study [54, 56–58, 60, 62, 64, 65, 67, 69, 75, 77]. In agreement with our observation, Hox genes are only expressed in the imaginal discs of the planktotrophic pilidium larva, and not during larval development [40]. Similarly, Hox genes are first expressed in the invagination-stage larva of the hoplonemertean *P. californiensis* [59]. On the other hand, the bifunctional protein βcatenin controls endoderm specification in the nemertean *Cerebratulus lacteus* [41], as it also happens in other bilaterian embryos [83–85]. These observations together indicate that some basic molecular aspects of the nemertean development have diverged compared with other spiralian lineages, in particular those controlling early embryogenesis. Whether these differences are based on gene innovation, such as the *bicoid* gene of dipterans [86, 87], or on the redeployment of factors and gene networks in new developmental contexts will require further analyses.

### The evolution of the adelphophagic intracapsular forms in the Pilidiophora

The presence of distinct larval types within the Pilidiophora—a group that shares a common mode of early development and a similar adult morphology—offers an ideal opportunity to address the developmental processes that underpin the evolution of new larval forms. The transition from a planktotrophic to a lecithotrophic pilidium is markedly related to the loss of the circumoral ciliated lobes of pelagic forms [28, 30–32], and the storage of yolk content in the larval epidermis [37]. From a developmental perspective, the juvenile still develops from two or three pairs of imaginal discs in lecithotrophic forms [30, 31], as in the typical planktotrophic pilidium [2, 17, 22]. However, there is a precocious development of the imaginal discs in lecithotrophic forms, which develop all the rudiments of the juvenile early and more or less simultaneously, instead of progressively as the larva feeds. All these developmental changes are observed in the intracapsular forms of *L. ruber* and *L. viridis* [29, 33–36] (this study), and are also well-established consequences of the transition from a planktotrophic to lecithotrophic nutritional mode in many other bilaterian lineages [88].

While the presence of intracapsular development is an obvious advantage in the intertidal habitat of *L. ruber* and *L. viridis*, the ecological impact of adelphophagy is not that clear. Moreover, how the adelphophagic behavior evolved in nemerteans, and which are—if any—the developmental changes associated with this event, is still unknown. Adelphophagy has been reported in several bilaterian groups, including vertebrates, echinoderms, insects, molluscs, annelids and platyhelminthes [89, 90], and often involves heterochronic shifts in the maturation of feeding structures [91, 92], changes in the morphology, composition and development of the nurturing-eggs [89, 93–95], and the development of specialized structures in the embryos [96–98]. Our study demonstrates that the ingestion of siblings in *L. ruber* starts before metamorphosis (Fig. 3), and extends during the whole time the post-metamorphic juveniles are inside the egg string. Moreover, predation is not only restricted to unviable or uncleaved eggs—oophagy—but can also occur with seemingly normal embryos (Fig. 6). These observations contrast with previous reports on *L. ruber* stating that adelphophagy was limited to oocytes and happened after metamorphosis [34]. Importantly, the larvae and metamorphosis of *L. ruber* and *L. viridis* seems to be largely similar. We did not observe any specific morphological adaptation of the Schmidt’s larva of *L. ruber* to a predatory behavior, such as a muscular pharynx or anchoring/collecting structures (Fig. 3). The only reported difference between the larval forms of *L. ruber* and *L. viridis* is the sealing of the mouth after metamorphosis in *L. viridis* [35]. Retaining an opened mouth was related to the post-metamorphic adelphophagy of *L. ruber* [37], but our results cast doubt on this interpretation, since the Schmidt’s larva of *L. ruber* uptakes yolk nutrient way before metamorphosis (Fig. 4). Therefore, the presence of adelphophagy in *L. ruber* seems to be neither related to the presence of specialized nurturing-eggs, since the larva also feeds on developed embryos, nor on the presence of any particular morphological or developmental adaptation. Further comparative work on *L. viridis* might help illuminate the evolution of this fascinating trait in nemertean embryos.

## Conclusions

In this study, we characterize the embryonic development of the adelphophagic intracapsular larva—Schmidt’s larva—of the nemertean worm *L. ruber*. A detailed morphological analysis showed that the Schmidt’s larva develops after gastrulation through the formation of two pairs of imaginal discs—cephalic and trunk pairs—a proboscis and a gut rudiment, and a thin epidermis, which is the only transitory tissue of the larva. The Schmidt’s larva of *L. ruber* actively feed on mostly unfertilized eggs, but also developing siblings. This intracapsular cannibalism is not associated with the formation of any specialized feeding structure or muscular tissue in the Schmidt’s larva, and might be mediated by the strong ciliation observed in the lining epithelium of the pharynx rudiment. Similar to other lecithotrophic pilidium larva, the formation of the imaginal discs of the Schmidt’s larva in *L. ruber* occurs simultaneously, and independently of the feeding on other eggs. Anterior (*foxQ2*, *six3/6*, *otx*, *gsc*) and posterior (*evx*, *cdx*) gene markers are expressed in the cephalic and trunk discs, respectively, and endomesodermal genes are detected in the pharynx and gut rudiments (*foxA*, *GATA456*-*a*) and broadly in all discs (*twi*-*a*). Therefore, the basic molecular patterning of the early juvenile is already established in the Schmidt’s larva. During organogenesis, the imaginal discs grow and fuse forming the epidermis of the juvenile, and differentiate into the basic definitive cell types and tissues. However, the final morphology of the worm is not attained until several days after metamorphosis. The only tissue that is discarded is the larval epidermis. Altogether, our results indicate that the developmental and morphological adaptations of the intracapsular larva of *L. ruber* are comparable to those observed in pelagic lecithotrophic pilidium forms, suggesting that similar evolutionary trajectories underpin the evolution of the great diversity of larval strategies observed in the Pilidiophora.

## Additional files

10.1186/s13227-015-0023-5 Adult *Lineus ruber* feeding on the annelid *Platynereis dumerilii.*

10.1186/s13227-015-0023-5 Analyses of gene orthology. (**A**–**G**) Maximum likelihood phylogenetic trees for *cdx*, *evx*, *foxQ2/foxA*, *GATA456*-*a*, *gsc/otx*, *six3/6*, and *twi*-*a*. Replicate bootstrap values were calculated with the autoMRE option in RAxML v.8. *Lineus ruber* sequences are highlighted in red. Models of protein evolution used for each tree: *cdx*: LG, *evx*: LG, *foxQ2/foxA*: LG, *GATA456*-*a*: Dayhoff, *gsc/otx*: LG, *six3/6*: LG, and *twi*-*a*: JTT.
10.1186/s13227-015-0023-5 Oviposition in *Lineus ruber.*

10.1186/s13227-015-0023-5 The embryonic development of *Lineus ruber* inside the egg masses. (**A**–**J**) Photographs of live specimens taken under the stereomicroscope. (**A**) At the moment of oviposition (0 days after oviposition, *dao*), the fertilized oocytes are packed inside pyriform capsules that attach to a central scaffold (arrowheads). The whole egg capsules are embedded in a jelly that isolates them from the exterior. Oocytes are rich in yolk content. (**B**) Cleavage takes about 5 days and results in the formation of a coeloblastula. At this stage, one can observe arrested embryos with abnormal patterns of cell division (arrow). (**C**) 9 days after oviposition, embryos have gastrulated (arrow) and show a conspicuous blastopore (asterisk). There are often several developing embryos together with arrested cleaving embryos (arrowhead) within the same egg capsule. (**D**) 12 days old embryos adopt the form of the early Schmidt’s larva (arrows). (**E**) The Schmidt’s larvae (arrows) feed on the arrested embryos present within the same egg capsule, growing in size and filling up the blind gut with nutrients. (**F–G**) The growth and differentiation of the imaginal discs of the Schmidt’s larva result in the formation of the juvenile worm (arrows). There are still unfertilized eggs (arrowhead) together with the metamorphic larvae within the same capsule. (**H**) The juveniles soon adopt a worm-like appearance, many of them hatching out of the capsule, but remaining within the jelly of the egg mass. (**I**–**J**) The juveniles stay inside the egg string for about two weeks more, while they mature and adopt an adult-like looking. After 30 days of development, pigmentation in the head region (arrowhead in **I** and in the inset in **J**), likely associated with the formation of eyes, becomes visible. Later on, the pigmentation extends to the rest of the body (see inset in **J**). About 40 days after oviposition, the fully mature juveniles (arrows in **J**) escape from the remains of the egg string. At this time, there are still some unfertilized eggs and less matured juveniles (arrow, inset in **J**). *hd* head, *iy* internalized yolk.
10.1186/s13227-015-0023-5 Developing embryos inside the egg capsule.
10.1186/s13227-015-0023-5 Hatching of *Lineus ruber* juveniles from the egg mass.
10.1186/s13227-015-0023-5 Hatchlings of *Lineus ruber.*

10.1186/s13227-015-0023-5 Juveniles of *Lineus ruber* feeding on *Platynereis dumerilii* eggs.
10.1186/s13227-015-0023-5 The dorsal side of the Schmidt’s larva. (**A**, **B**) z projections of confocal scans of a larva labeled against F-actin (gray) counterstained with the nuclear marker Sytox Green (yellow) 10 days after oviposition. (**A**) The Schmidt’s larva shows an accumulation of mesenchymal cells in between the cephalic discs and the pharynx rudiment (white arrowheads). However, there are no conspicuous epithelial discs that can be assigned as cerebral organ discs at this stage. (**B**) On the dorsal side of the Schmidt’s larva, there is no obvious unpaired dorsal disc, but scattered mesenchymal cells (white arrowheads) located just underneath the larval epidermis. (**A**, **B**) are dorsal views. In both panels, anterior is to the left. bg, blind gut; cd, cephalic discs; le, larval epidermis; pr, pharynx rudiment; td, trunk discs. Scale bars, 50 μm in both panels.
10.1186/s13227-015-0023-5 Expression of *gsc* in mature juveniles. (**A′**–**A′′′**) z projection of a whole-mount in situ hybridization of *gsc* in an early juvenile counterstained with the nuclear marker DAPI (red). Signal is observed in the epidermal invaginations that correspond to the cerebral organ canals (inset in **A′′**; squares in **A′** and **A′′** indicate the area magnified). (**B′**, **B′′**) Whole-mount colorimetric in situ hybridization of *gsc* in mature juveniles of L. ruber. At this stage, *gsc* is expressed in a dorsal anterior domain (arrow) and in two internal paired domains at the end of the cephalic slit, where the cerebral organs are located (arrowheads). (**A′**–**A′′′**) is a dorsolateral view. (**B′**) is a ventral view. (**B′′**) is a lateral view. In all panels, anterior to the left. cs, cephalic slits; mo, mouth.

## Abbreviations

DAPI: 4 ‘,6-diamino-2-fenilindol
EdU: 5-ethynyl-2′-deoxyuridine
