# Supplementary figures and images for: Evolution and development of the adelphophagic, intracapsular Schmidt’s larva of the nemertean Lineus ruber
Source: EvoDevo. 2015 Sep 28;6:28. doi: 10.1186/s13227-015-0023-5 (PMC4584431; doi:10.1186/s13227-015-0023-5)

A

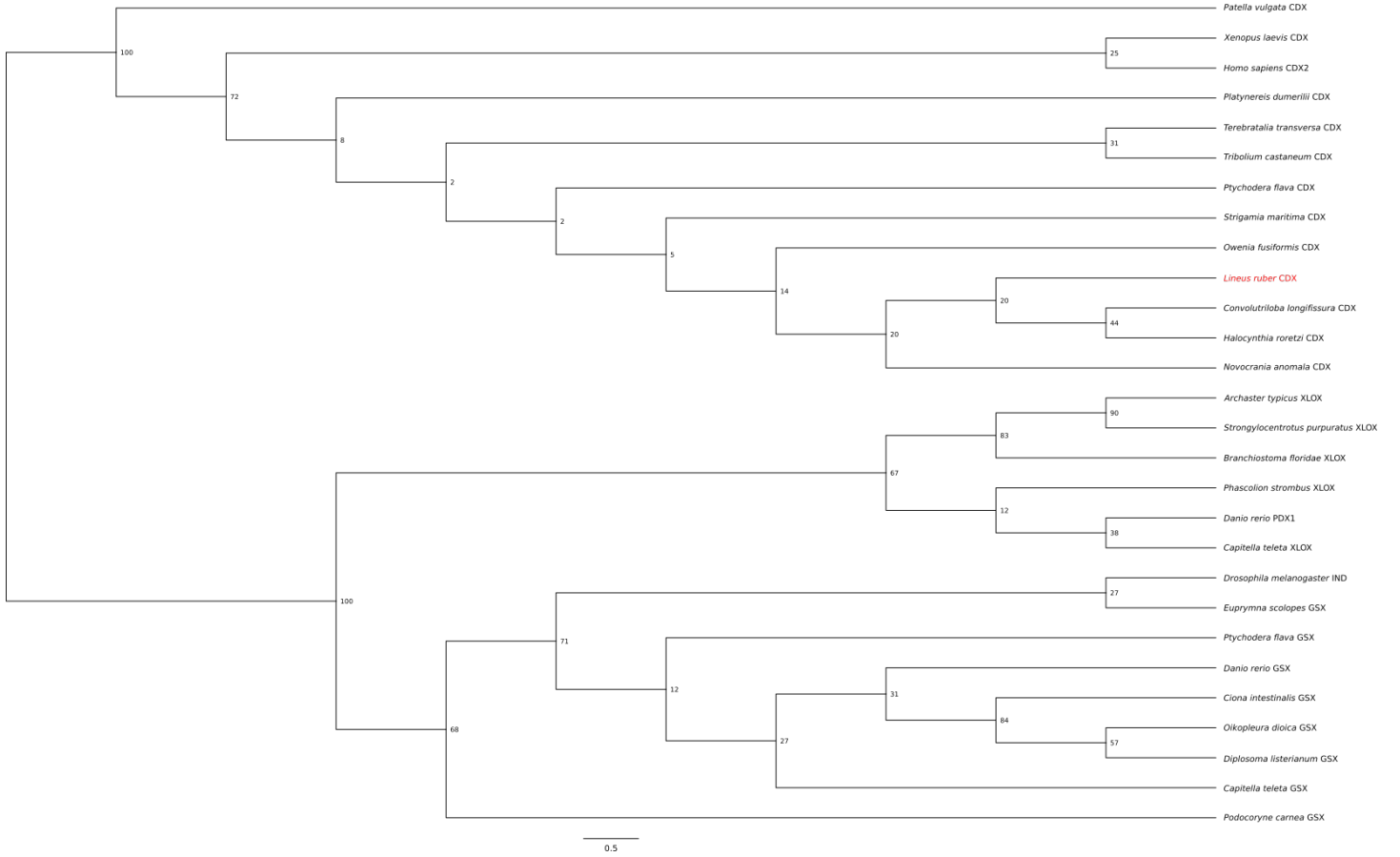

B

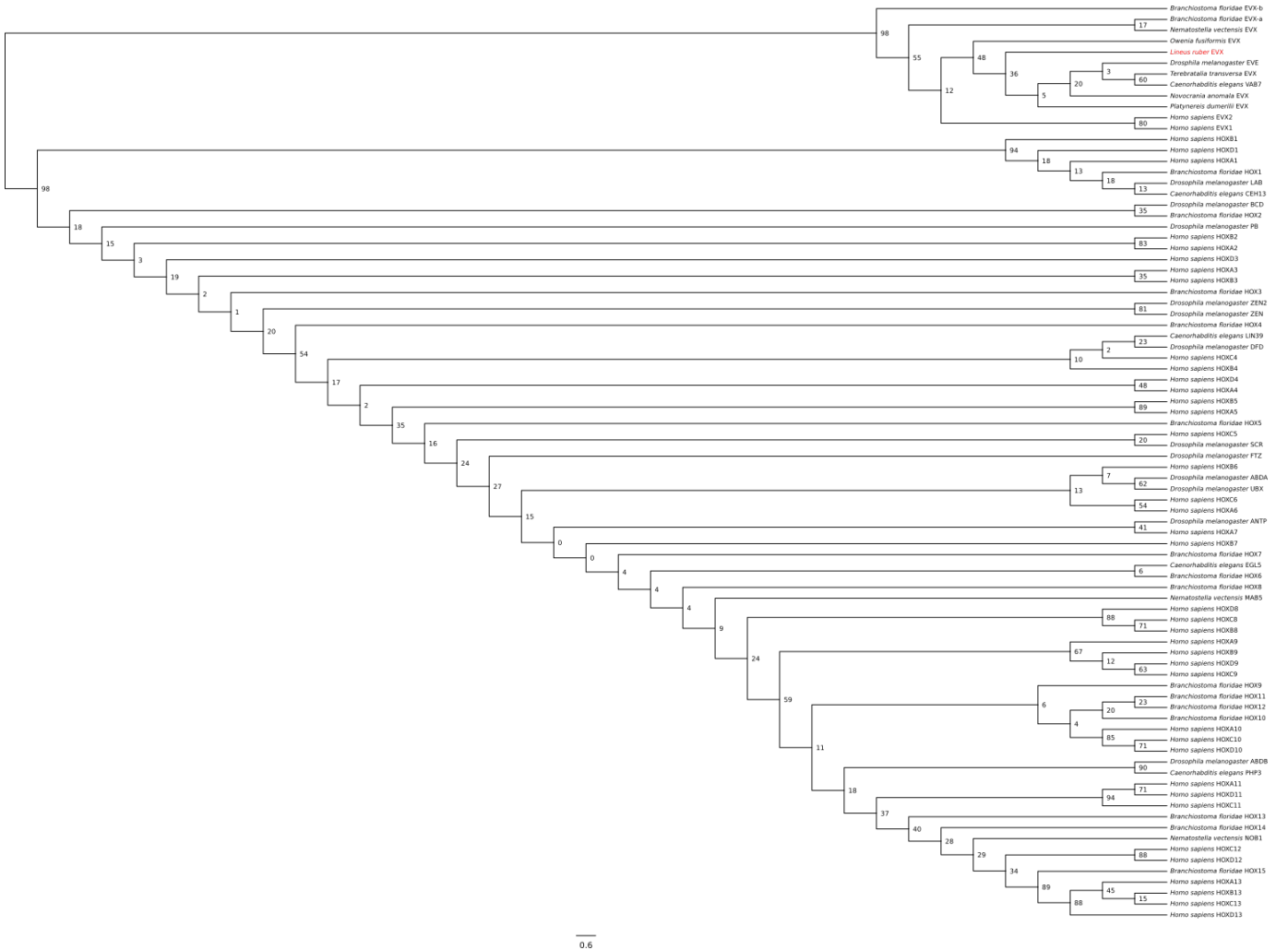

C

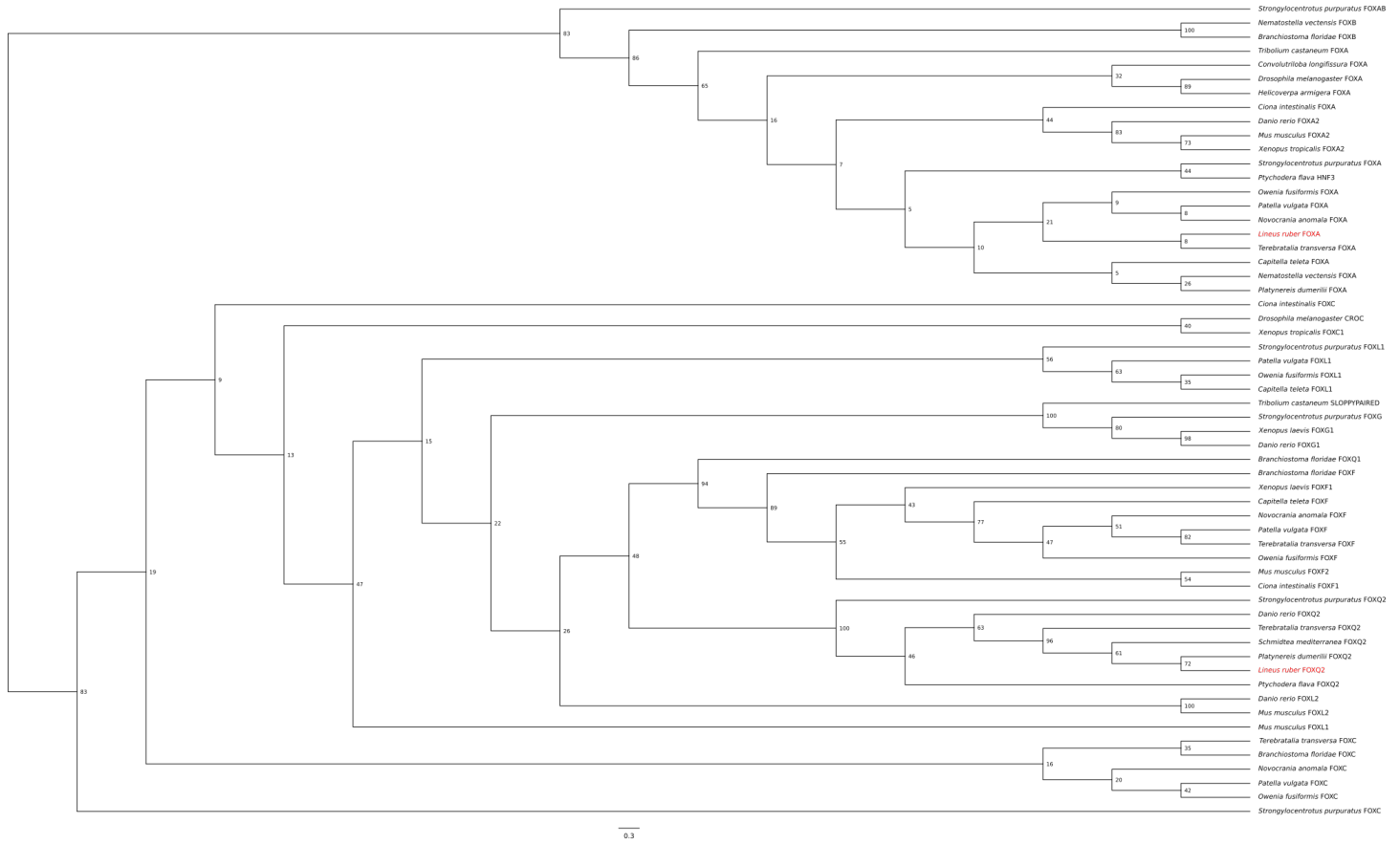

D

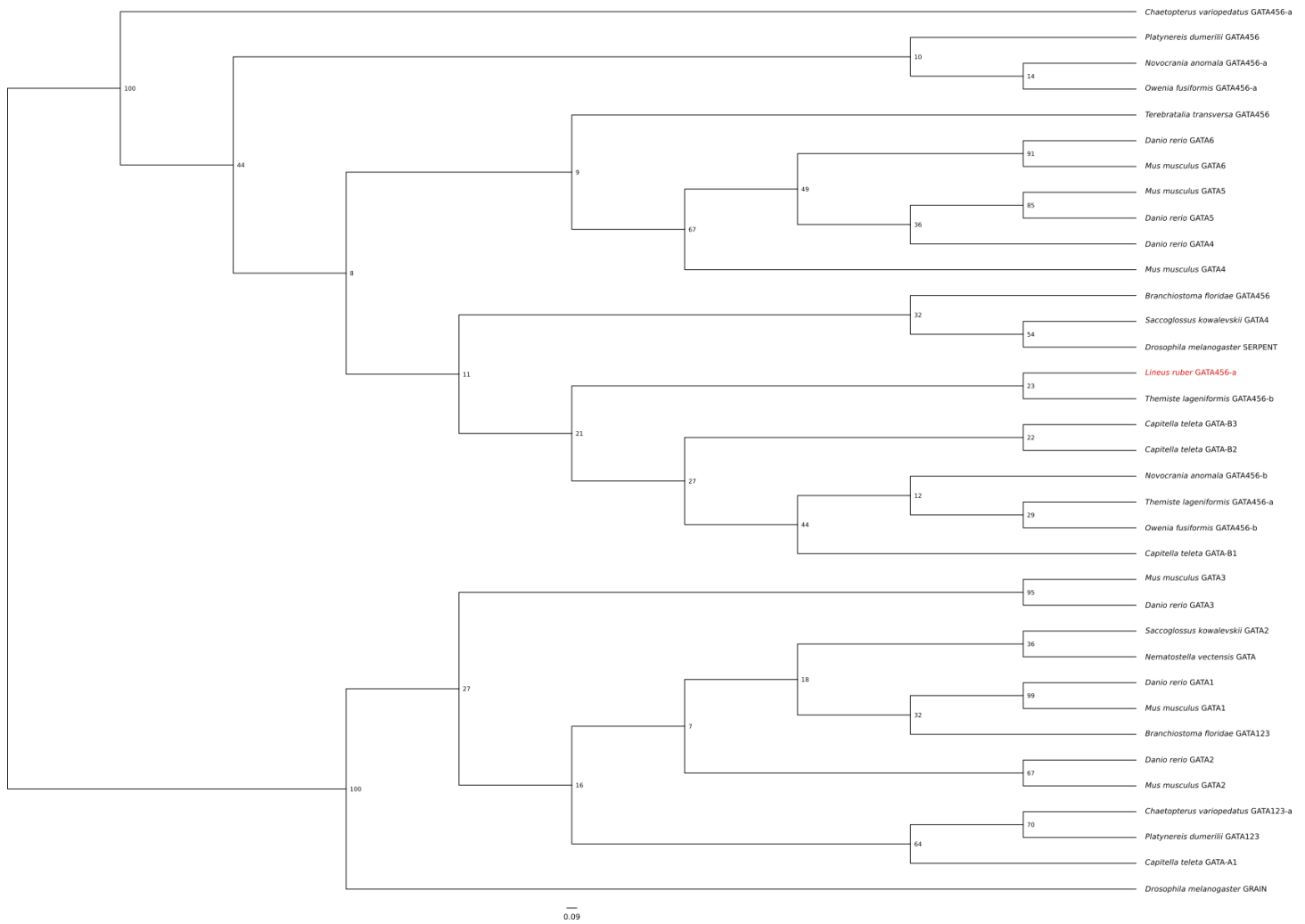

E

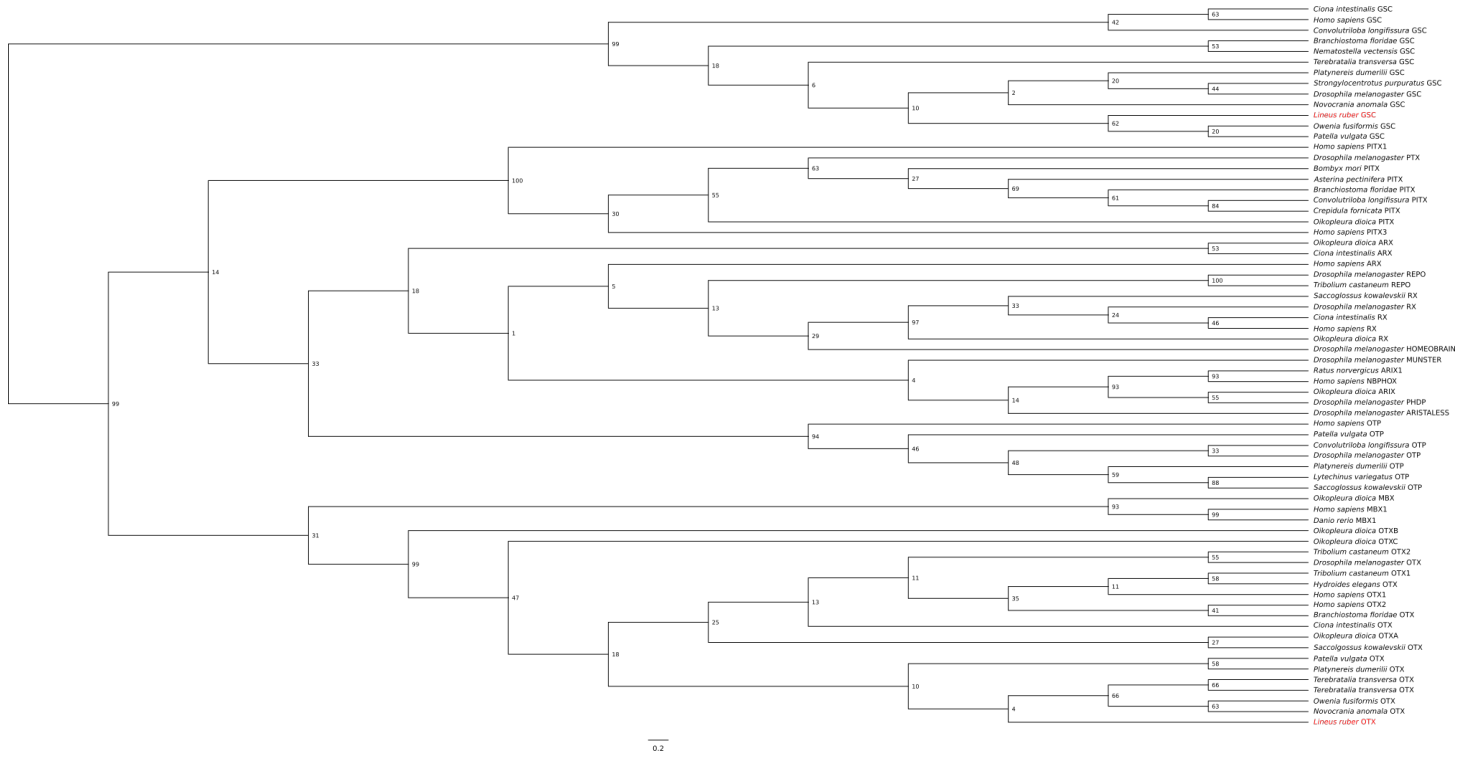

F

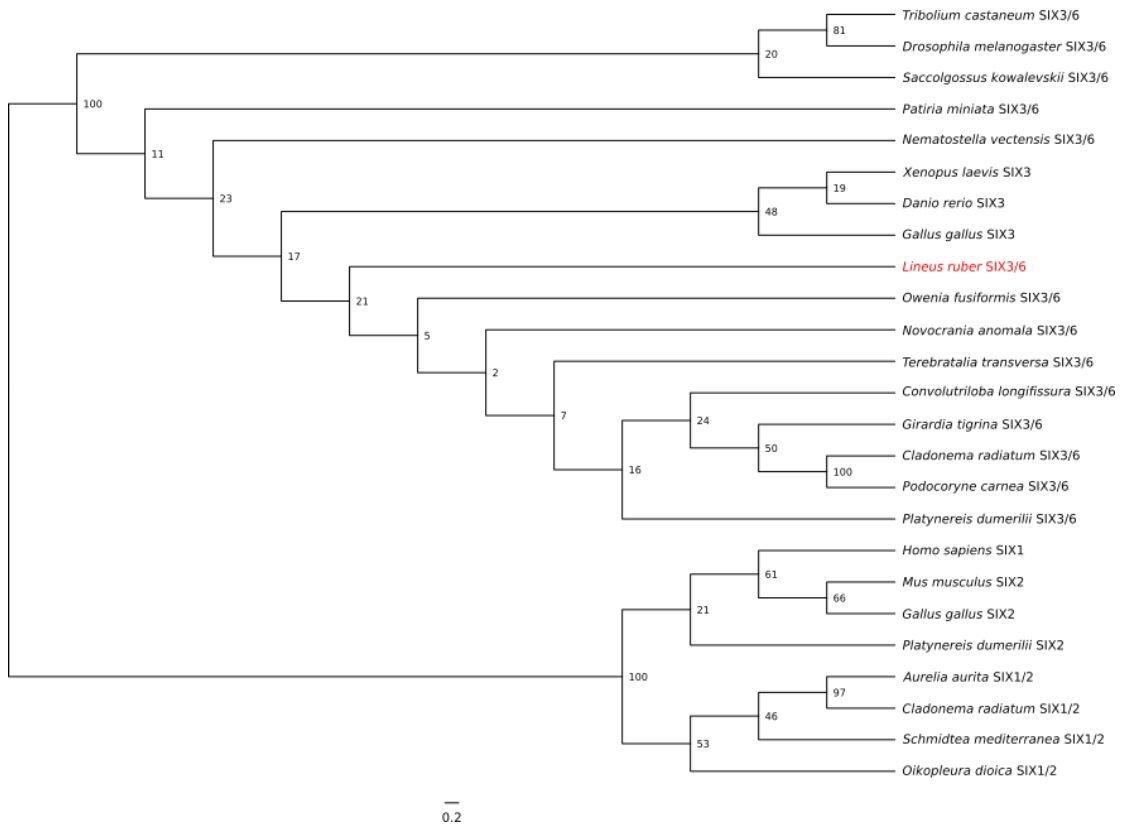

G

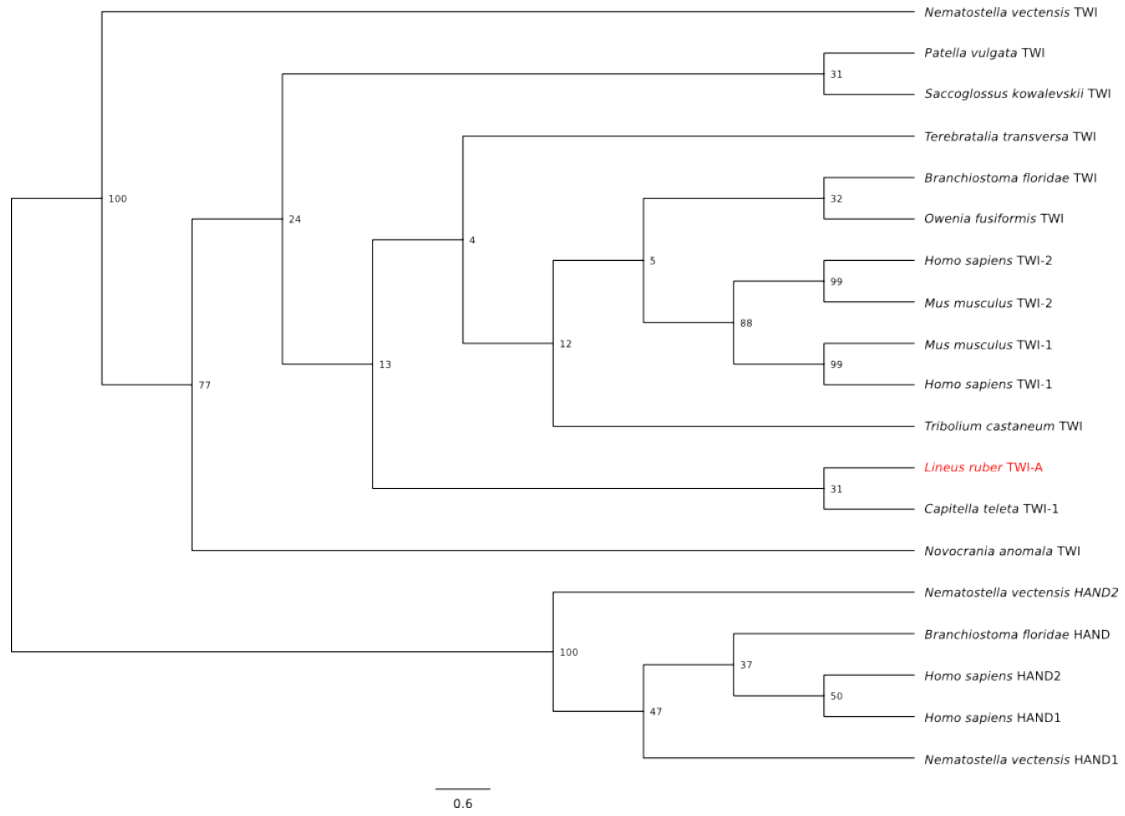

Supplement: Supplementary file 2 — 10.1186/s13227-015-0023-5 Analyses of gene orthology. (A–G) Maximum likelihood phylogenetic trees for cdx, evx, foxQ2/foxA, GATA456-a, gsc/otx, six3/6, and twi-a. Replicate bootstrap values were calculated with the autoMRE option in RAxML v.8. Lineus ruber sequences are highlighted in red. Models of protein evolution used for each tree: cdx: LG, evx: LG, foxQ2/foxA: LG, GATA456-a: Dayhoff, gsc/otx: LG, six3/6: LG, and twi-a: JTT. [file 13227_2015_23_MOESM2_ESM.pdf]

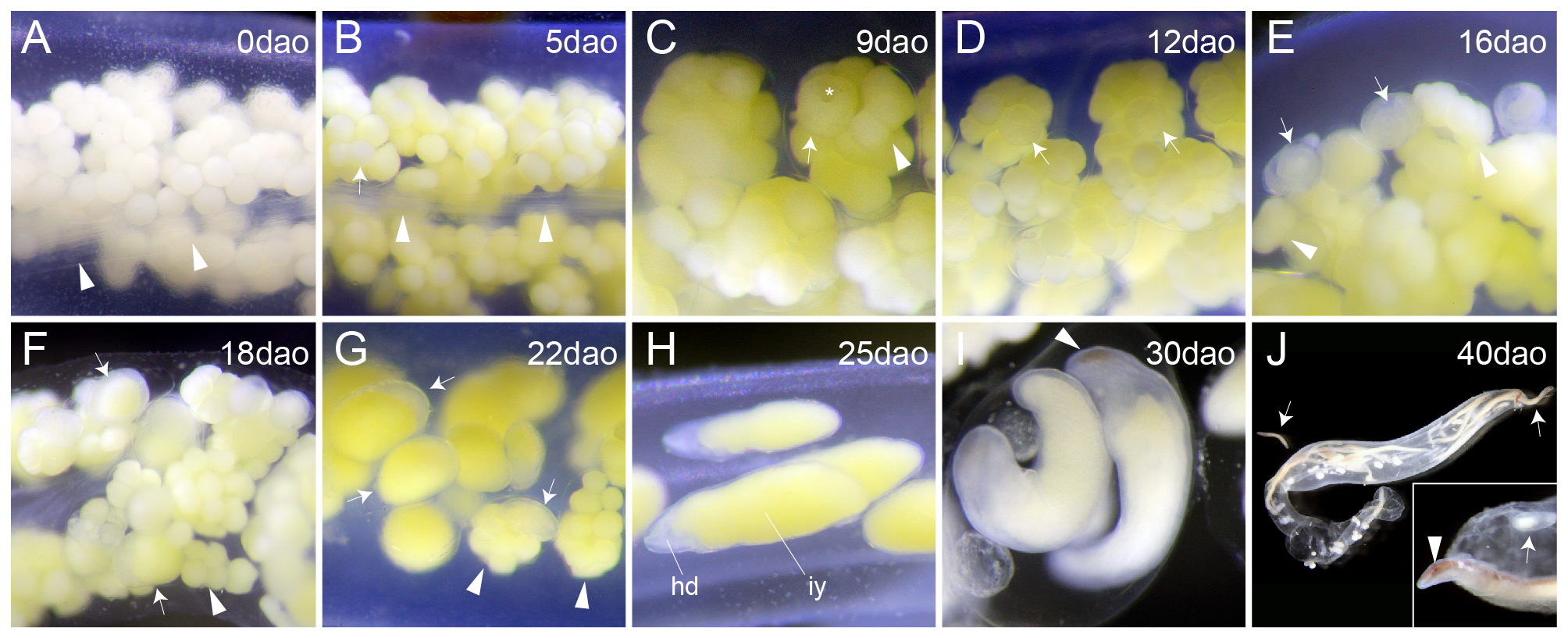

Supplement: Supplementary file 4 — 10.1186/s13227-015-0023-5 The embryonic development of Lineus ruber inside the egg masses. (A–J) Photographs of live specimens taken under the stereomicroscope. (A) At the moment of oviposition (0 days after oviposition, dao), the fertilized oocytes are packed inside pyriform capsules that attach to a central scaffold (arrowheads). The whole egg capsules are embedded in a jelly that isolates them from the exterior. Oocytes are rich in yolk content. (B) Cleavage takes about 5 days and results in the formation of a coeloblastula. At this stage, one can observe arrested embryos with abnormal patterns of cell division (arrow). (C) 9 days after oviposition, embryos have gastrulated (arrow) and show a conspicuous blastopore (asterisk). There are often several developing embryos together with arrested cleaving embryos (arrowhead) within the same egg capsule. (D) 12 days old embryos adopt the form of the early Schmidt’s larva (arrows). (E) The Schmidt’s larvae (arrows) feed on the arrested embryos present within the same egg capsule, growing in size and filling up the blind gut with nutrients. (F–G) The growth and differentiation of the imaginal discs of the Schmidt’s larva result in the formation of the juvenile worm (arrows). There are still unfertilized eggs (arrowhead) together with the metamorphic larvae within the same capsule. (H) The juveniles soon adopt a worm-like appearance, many of them hatching out of the capsule, but remaining within the jelly of the egg mass. (I–J) The juveniles stay inside the egg string for about two weeks more, while they mature and adopt an adult-like looking. After 30 days of development, pigmentation in the head region (arrowhead in I and in the inset in J), likely associated with the formation of eyes, becomes visible. Later on, the pigmentation extends to the rest of the body (see inset in J). About 40 days after oviposition, the fully mature juveniles (arrows in J) escape from the remains of the egg string. At this time, there are st [file 13227_2015_23_MOESM4_ESM.jpg]

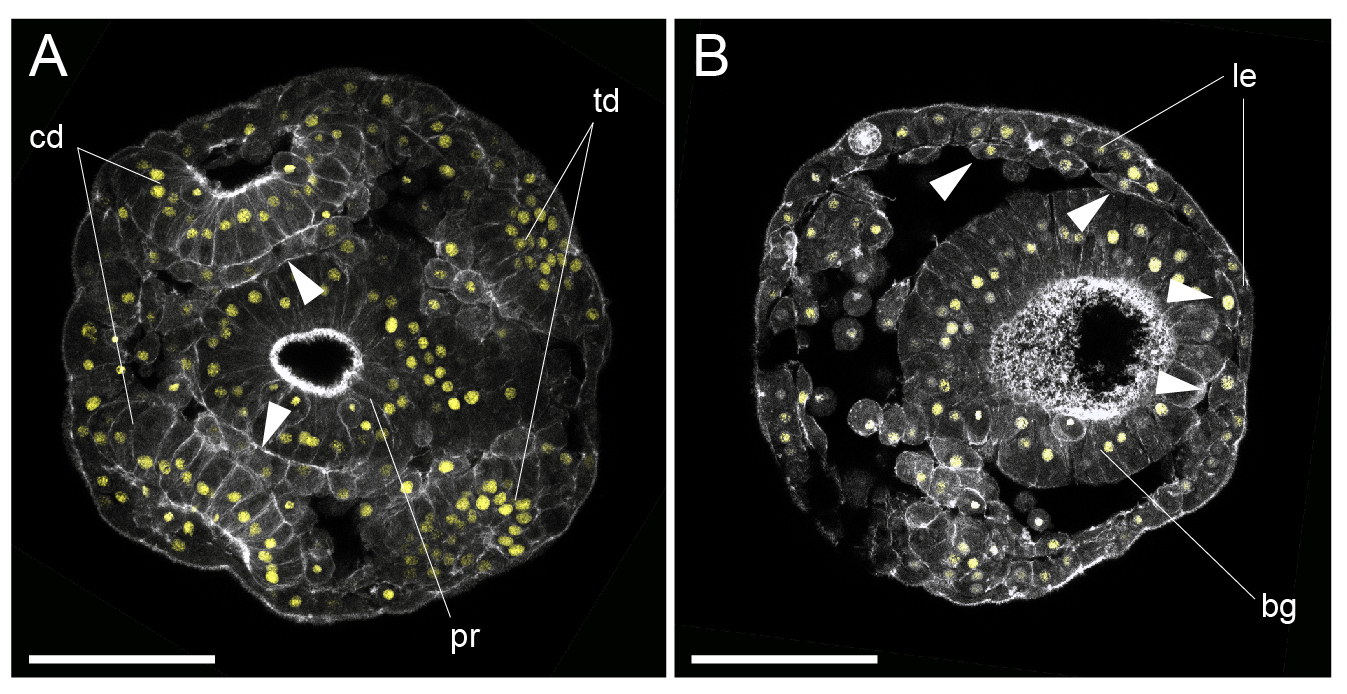

Supplement: Supplementary file 9 — 10.1186/s13227-015-0023-5 The dorsal side of the Schmidt’s larva. (A, B) z projections of confocal scans of a larva labeled against F-actin (gray) counterstained with the nuclear marker Sytox Green (yellow) 10 days after oviposition. (A) The Schmidt’s larva shows an accumulation of mesenchymal cells in between the cephalic discs and the pharynx rudiment (white arrowheads). However, there are no conspicuous epithelial discs that can be assigned as cerebral organ discs at this stage. (B) On the dorsal side of the Schmidt’s larva, there is no obvious unpaired dorsal disc, but scattered mesenchymal cells (white arrowheads) located just underneath the larval epidermis. (A, B) are dorsal views. In both panels, anterior is to the left. bg, blind gut; cd, cephalic discs; le, larval epidermis; pr, pharynx rudiment; td, trunk discs. Scale bars, 50 μm in both panels. [file 13227_2015_23_MOESM9_ESM.jpg]

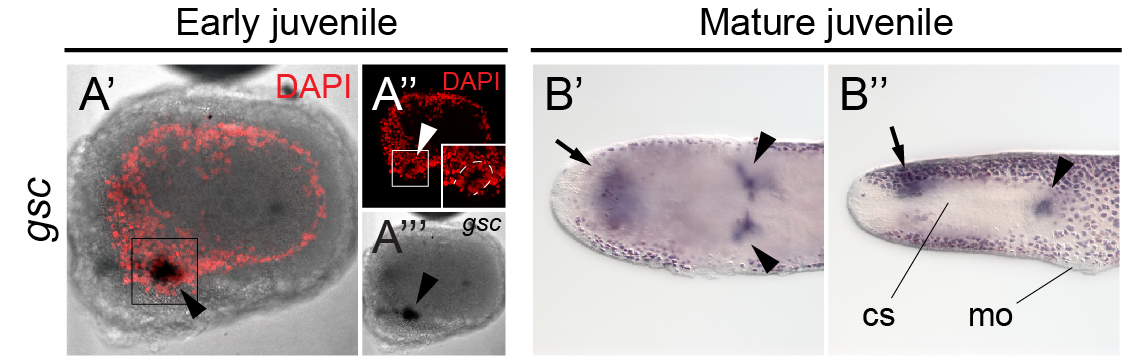

Supplement: Supplementary file 10 — 10.1186/s13227-015-0023-5 Expression of gsc in mature juveniles. (A′–A′′′) z projection of a whole-mount in situ hybridization of gsc in an early juvenile counterstained with the nuclear marker DAPI (red). Signal is observed in the epidermal invaginations that correspond to the cerebral organ canals (inset in A′′; squares in A′ and A′′ indicate the area magnified). (B′, B′′) Whole-mount colorimetric in situ hybridization of gsc in mature juveniles of L. ruber. At this stage, gsc is expressed in a dorsal anterior domain (arrow) and in two internal paired domains at the end of the cephalic slit, where the cerebral organs are located (arrowheads). (A′–A′′′) is a dorsolateral view. (B′) is a ventral view. (B′′) is a lateral view. In all panels, anterior to the left. cs, cephalic slits; mo, mouth. [file 13227_2015_23_MOESM10_ESM.jpg]
